# Methylation of histone H3 lysine 4 is required for maintenance of beta cell function in adult mice

**DOI:** 10.1007/s00125-023-05896-6

**Published:** 2023-03-13

**Authors:** Ben Vanderkruk, Nina Maeshima, Daniel J. Pasula, Meilin An, Cassandra L. McDonald, Priya Suresh, Dan S. Luciani, Francis C. Lynn, Brad G. Hoffman

**Affiliations:** 1 Diabetes Research Group, British Columbia Children’s Hospital Research Institute, Vancouver, BC Canada; 2 Department of Surgery, University of British Columbia, Vancouver, BC Canada

**Keywords:** Beta cell, Chromatin, COMPASS, DPY30, H3K4me3, Insulin, Transcription, Type 2 diabetes

## Abstract

**Aims/hypothesis:**

Beta cells control glucose homeostasis via regulated production and secretion of insulin. This function arises from a highly specialised gene expression programme that is established during development and then sustained, with limited flexibility, in terminally differentiated cells. Dysregulation of this programme is seen in type 2 diabetes but mechanisms that preserve gene expression or underlie its dysregulation in mature cells are not well resolved. This study investigated whether methylation of histone H3 lysine 4 (H3K4), a marker of gene promoters with unresolved functional importance, is necessary for the maintenance of mature beta cell function.

**Methods:**

Beta cell function, gene expression and chromatin modifications were analysed in conditional *Dpy30* knockout mice, in which H3K4 methyltransferase activity is impaired, and in a mouse model of diabetes.

**Results:**

H3K4 methylation maintains expression of genes that are important for insulin biosynthesis and glucose responsiveness. Deficient methylation of H3K4 leads to a less active and more repressed epigenome profile that locally correlates with gene expression deficits but does not globally reduce gene expression. Instead, developmentally regulated genes and genes in weakly active or suppressed states particularly rely on H3K4 methylation. We further show that H3K4 trimethylation (H3K4me3) is reorganised in islets from the *Lepr*^*db/db*^ mouse model of diabetes in favour of weakly active and disallowed genes at the expense of terminal beta cell markers with broad H3K4me3 peaks.

**Conclusions/interpretation:**

Sustained methylation of H3K4 is critical for the maintenance of beta cell function. Redistribution of H3K4me3 is linked to gene expression changes that are implicated in diabetes pathology.

**Graphical abstract:**

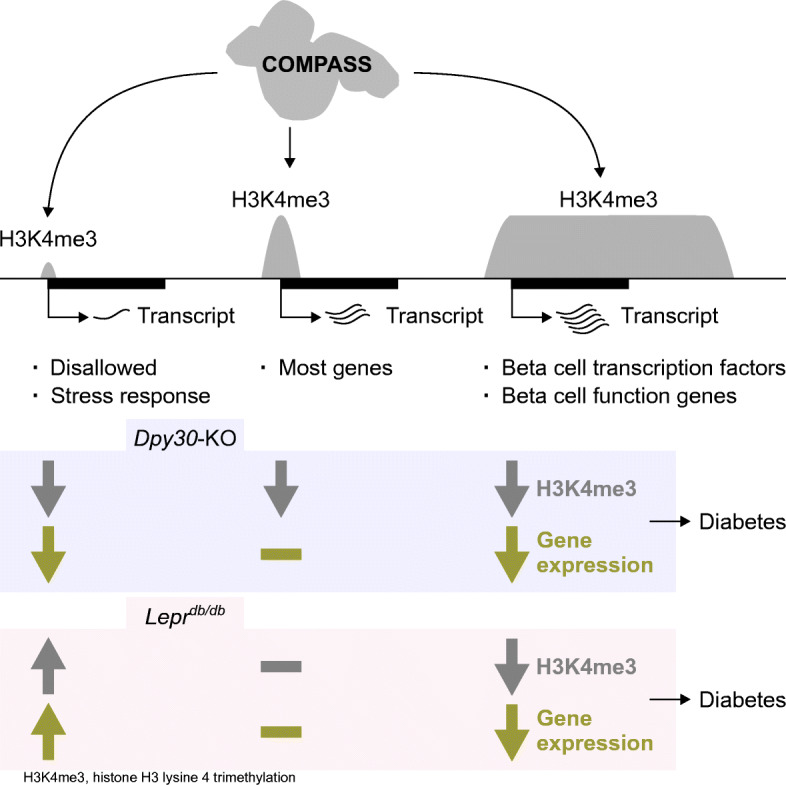

**Supplementary Information:**

The online version of this article (10.1007/s00125-023-05896-6) contains peer-reviewed but unedited supplementary material.

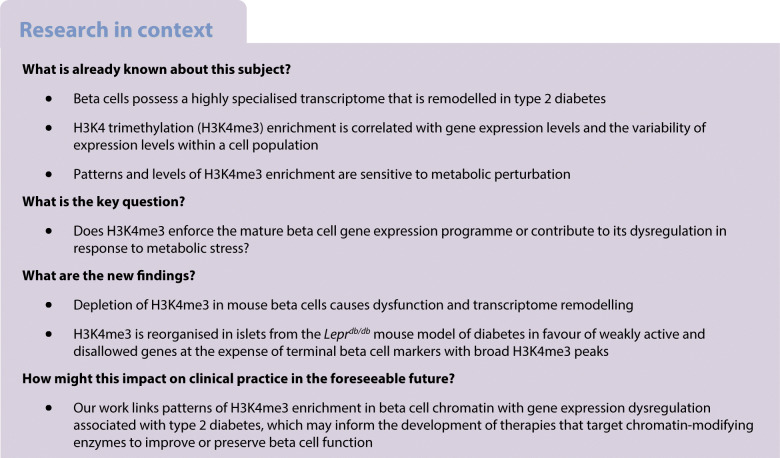



## Introduction

Tissue-specific transcription programmes rely on precise activation and maintenance of specific genes and stable repression of other genes. Chromatin modifications are central to this system of cellular memory. Beta cells exposed to metabolic stress show widespread changes in gene expression, including activation of developmentally silenced genes and decreased expression of genes that are important for insulin biosynthesis or glucose responsiveness or that enforce cell identity [1, 2]. There is mounting evidence that epigenetic mechanisms maintain mature beta cell function and identity and that the chromatin landscape is reshaped in beta cells exposed to chronic metabolic stress [3–9]. Chromatin-modifying enzymes are therefore promising therapeutic targets. For example, inhibition of histone H3 lysine 4 (H3K4) methyltransferases has been suggested to prevent ectopic gene activation [4] or increase replication [10] in beta cells during type 2 diabetes; however, the consequences of such a strategy are unknown.

Methylation of H3K4 is a highly conserved post-translational modification linked to RNA polymerase II-dependent transcription. H3K4 trimethylation (H3K4me3) is reliably enriched at active promoters, where the level of enrichment correlates with transcriptional output [11]. H3K4 monomethylation (H3K4me1) is enriched at active and primed enhancers, promoters and gene bodies [12]. The extent to which H3K4 methylation regulates transcription is less clear. On the one hand, H3K4 methylation can regulate gene activity by recruitment of transcriptional machinery [13] and chromatin remodellers [14], facilitating enhancer–promoter interactions [15], and by blocking repression [16, 17]. On the other hand, global (i.e. genome-wide) ablation of H3K4 methylation is remarkably well tolerated [18–20]. Evidence in animal models suggests that H3K4 methyltransferases impact the expression of only a fraction of genes and are necessary only for specific roles in development or in response to environmental stress [20–23]. Further, loss of H3K4 methylation is associated with gene upregulation in some circumstances [24, 25]. Rather than being a strict requirement for gene activation, the emerging view is that H3K4 methylation fine-tunes gene expression by providing a context-specific but generally activating signal [26], reducing variability of transcription [27], providing a persistent memory of transcriptional activity [28] and maintaining the potential for future expression [29]. H3K4 methylation also has a non-transcriptional role in DNA damage repair [30]. Thus, while H3K4me3 is predictive of transcription [31], the consequences of its removal are difficult to predict.

Mono-, di- and trimethylation of H3K4 are catalysed by six partially redundant multiprotein complexes distinguished by the central methyltransferase: mixed lineage leukaemia protein-1 to -4 (MLL1, MLL2, MLL3, MLL4) and SET domain containing 1A and 1B (SETD1A and SETD1B). These complexes, called the COMPASS (complex of proteins associated with Set1) family, influence gene activity via distinct catalytic and non-catalytic functions. Recruitment of COMPASS by a variety of factors directs methylation of H3K4 at particular genomic loci [32]. The COMPASS family can also recruit a variety of factors themselves, including cell cycle regulators, tumour suppressors and other chromatin-modifying enzymes [33]. The biological relevance of these interactions is exemplified by reports that cells expressing catalytically inactive mutants of methyltransferase subunits display milder defects to gene expression and cell functions than cells completely lacking the same subunit [18, 34–36]. Non-catalytic subunits of COMPASS vary but always include a core subcomplex of WD repeat-containing protein 5 (WDR5), retinoblastoma-binding protein 5 (RBBP5), absent, small or homeotic 2-like protein (ASH2L) and Dumpy-30 (DPY30) [37]. WDR5, RBBP5 and ASH2L are required for complex stability and enzymatic activity [38]. DPY30 is not required for complex stability or methyltransferase activity but improves coordination of the COMPASS catalytic domain with H3K4 in the context of a complete nucleosome [39]. For this reason, DPY30 is required for methylation of H3K4 genome-wide in vivo [39]. *DPY30* and the other core subunits are essential genes [40] but, among 27 tissues tested in the Human Protein Atlas tissue-specific transcriptome project, the median expression of each was lowest in the pancreas [41]. DPY30 is enriched in islets compared with pancreatic exocrine cells in mice [42], indicating the potential relevance of COMPASS activity to pancreatic endocrine functions. Accordingly, we previously demonstrated that *Dpy30* fine-tunes cell fate decisions during mouse pancreas development in favour of endocrine lineages [23] and contributes to endocrine cell maturation [43]. By contrast, it is not known whether the continuous activity of H3K4 methyltransferases is necessary in terminally differentiated cells, where lineage-specific transcription programmes have already been established. We therefore sought to define the role of H3K4 methylation in the regulation of gene expression in terminally differentiated beta cells.

## Methods

### Animals

The following mice strains were used: *Dpy30*^*flox/flox*^ [23], *Rosa26*^*mTmG*^ (The Jackson Laboratory, USA; #007576 [44]), *Pdx1CreER*^*Tg*^ (The Jackson Laboratory; #024968 [45]), *Ins1*^*Cre*^ (The Jackson Laboratory; #026801 [46]), BKS *Lepr*^*db/db*^ and *Lepr*^*+/+*^ (The Jackson Laboratory; #000642). Genotypes used for *Pdx1CreER* studies were as follows—knockout: *Pdx1CreER*^*Tg/0*^;*Dpy30*^*flox/flox*^;*Rosa26*^*mTmG/+*^, wild-type: *Pdx1CreER*^*0/0*^;*Dpy30*^*flox/flox*^;*Rosa26*^*mTmG/+*^, or, if sorting recombined cells was required: *Pdx1CreER*^*Tg/0*^;*Dpy30*^*+/+*^;*Rosa26*^*mTmG/+*^. Regardless of genotype, all mice used in *Pdx1CreER* studies were administered 8 mg tamoxifen (Sigma-Aldrich, USA) by oral gavage three times over 5 days at 8 weeks of age and analysed 45 days later unless otherwise specified. Genotypes used for *Ins1*^*Cre*^ studies were as follows—knockout: *Ins1*^*Cre/+*^;*Dpy30*^*flox/flox*^, wild-type: *Ins1*^*+/+*^;*Dpy30*^*flox/flox*^, heterozygous: *Ins1*^*Cre/+*^; *Dpy30*^*flox/+*^. Mice in *Ins1*^*Cre*^ studies were analysed at 5 weeks unless otherwise specified. *Lepr*^*db/db*^ and *Lepr*^*+/+*^ mice were analysed at 12 weeks. Mice were kept under conventional conditions on a 12 h light/dark cycle with free access to water and food (Teklad 2918, Envigo, UK). Experiments were restricted to male mice of the indicated ages and genotypes; no other exclusion criteria were considered. Mice were not randomised and experimenters were not blinded to genotypes. Experiments were approved by the University of British Columbia Animal Care Committee (certificates A17-0045 and A18-0111).

Unfasted blood glucose was measured from the tail tip between 10:00 and 12:00 using a OneTouch Ultra Mini Glucometer (Johnson & Johnson, USA). For glucose tolerance tests, 2 g/kg body weight of glucose was injected intraperitoneally following a 6 h fast. Blood was sampled from a lateral saphenous vein. Serum was prepared by centrifuging blood samples at 9000 *g* for 9 min at 4°C. Serum insulin was measured using an ELISA (Alpco, USA).

Islets were isolated using collagenase digestion [43] and dispersed to single cells [47] as described previously. EGFP-positive tdTomato-negative cells were enriched from *Pdx1CreER*^*Tg/0*^;*Rosa26*^*mTmG*^ mice using a FACSAria II Cell Sorter (BD Biosciences, USA).

*Drosophila* S2 cells (Life Technologies, USA) were cultured at 25°C in Schneider’s *Drosophila* medium (Life Technologies) supplemented with 10% (vol/vol) heat-inactivated FBS (Life Technologies).

### Co-immunoprecipitation, immunoblotting and immunohistochemistry

Immunoprecipitation from islet cell nuclear lysates, immunoblotting of islet lysates and immunofluorescence analyses of paraffin-embedded pancreas sections were carried out using standard protocols as outlined in the electronic supplementary material (ESM) Methods using the antibodies listed in ESM Table 1.

### Chromatin immunoprecipitation–sequencing (ChIP-seq)

For *Pdx1CreER*^*Tg/0*^;*Dpy30*^*+/+*^; *Rosa26*^*mTmG/+*^ and *Pdx1CreER*^*Tg/0*^;*Dpy30*^*flox/flox*^;*Rosa26*^*mTmG/+*^ mice, 100,000 beta cells were pooled with 50,000 *Drosophila* S2 cells by FACS. For *Lepr*^*db/db*^ and *Lepr*^*+/+*^ mice, 100,000 dispersed islet cells were counted using a hemacytometer and spiked with 50,000 *Drosophila* S2 cells without sorting. The ULI-NChIP procedure [48] was used to generate ChIP-seq libraries using the antibodies listed in ESM Table 1 in biological duplicate. Immunoprecipitated DNA was prepared for sequencing using the NEBNext Ultra II DNA Library Prep Kit (New England Biolabs, USA). See ESM Methods for further details.

### RNA-seq

Beta cells (70,000–226,000 per mouse, *n*=3) from *Pdx1CreER*^*Tg/0*^;*Dpy30*^*+/+*^;*Rosa26*^*mTmG/+*^ and *Pdx1CreER*^*Tg/0*^;*Dpy30*^*flox/flox*^;*Rosa26*^*mTmG/+*^ mice were purified by FACS and spiked with 10% *Drosophila* S2 cells. mRNA isolation and library preparation were performed as described previously [43]. See ESM Methods for further details.

### Single-cell RNA-seq

Islet cells from one *Pdx1CreER*^*Tg/0*^;*Dpy30*^*+/+*^;*Rosa26*^*mTmG/+*^ mouse and one *Pdx1CreER*^*Tg/0*^;*Dpy30*^*flox/flox*^;*Rosa26*^*mTmG/+*^ mouse were processed through the Chromium Single Cell 3′ protocol using the Chromium Controller with Reagent Kit v3.1 and Dual Index Kit TT Set A (10x Genomics, USA). See ESM Methods for further details.

### Pyrosequencing

DNA from 100 islets was bisulphite converted using the EZ DNA Methylation-Direct Kit (Zymo Research, USA). Genomic regions were amplified using the PyroMark PCR Kit with CpG Assay primers Mm_Igf2_04_PM, Mm_Cd81_01_PM or Mm_Cdkn1c_01_PM (Qiagen, Germany). Per cent methylation at amplified CpGs was measured using the PyroMark Q96 MD (Qiagen).

### Electron microscopy

Islets were fixed in 2% (wt/vol) glutaraldehyde (Sigma-Aldrich) and then submitted to the Electron Microscopy Facility at McMaster University (Canada). Islets were post-fixed with 1% (wt/vol) osmium tetroxide, dehydrated in ethanol, embedded in Spurr’s resin and sectioned with a Leica UCT ultramicrotome. Sections were stained with uranyl acetate and lead citrate and imaged with a JEM 1200 EX TEMSCAN transmission electron microscope (JEOL, USA). Insulin granules were quantified using ImageJ v1.52a [49].

### Islet functional assays

Insulin secretion assays [43] and calcium imaging [50] were performed as described previously. Respiration was measured in dispersed islets using the Seahorse XF24 Extracellular Flux Analyzer (Agilent, USA).

### Analysis of publicly available data

Fastq files from GSE50244 [51], GSE50386 [52], GSE107489 [53] and GSE124742 [54] were downloaded from the Sequence Read Archive and analysed as described above. A table of per cent methylation of each cytosine in beta cells from 16- to 20-month-old C57BL6 mice was downloaded from GSE68618 [55] and converted to the GRCm38/mm10 reference genome using the University of California Santa Cruz liftover utility [56].

### Statistics

Bar plots show means ± SD with individual data points. In box and whisker plots, the central horizontal line indicates the median, the upper and lower limits of the box indicate the first and third quartiles, and the whiskers span 1.5× the IQR. Correlation was estimated using Spearman’s coefficient. *P* values were calculated using unpaired Welch’s *t* tests, Wilcoxon tests, Wald tests, mixed-effect models, ANOVA, Fisher’s exact tests or permutation tests as indicated. The Benjamini–Hochberg correction was applied where indicated. Calculations were performed in R v4.2.1 (http://www.r-project.org/) or Prism v9.5.0 (GraphPad Software, USA).

## Results

### Reduction of H3K4 methylation in beta cells of adult mice leads to glucose intolerance and hyperglycaemia

**Fig. 1:**
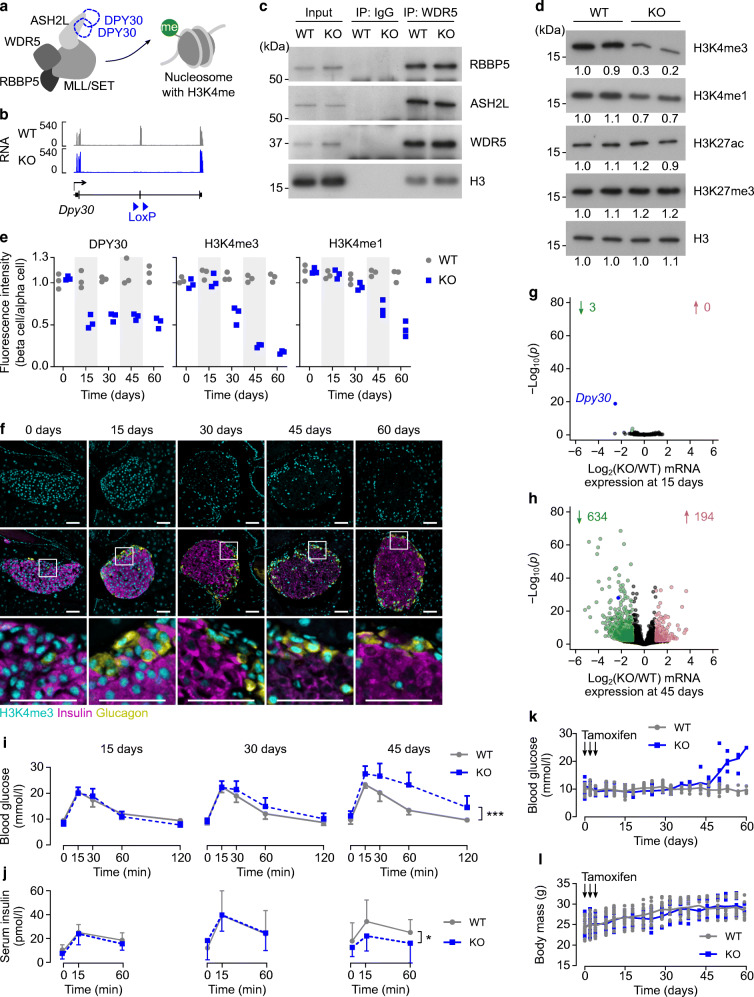
Reduction of H3K4 methylation in beta cells of adult mice leads to glucose intolerance and hyperglycaemia. (**a**) Schematic showing the core COMPASS subunits and a nucleosome methylated on H3K4. (**b**) Genome-aligned RNA-seq reads at the *Dpy30* gene locus in *Dpy30*-WT and *Dpy30*-KO beta cells 15 days post tamoxifen. Note that the floxed exon 4 is efficiently deleted in the KO cells. (**c**) Immunoblots showing the COMPASS subunits RBBP5, ASH2L and WDR5 and the nucleosome protein histone H3, co-immunoprecipitated with WDR5 or an IgG control from *Dpy30*-WT and *Dpy30*-KO islet cell nuclei 45 days post tamoxifen administration. Representative immunoblots of three independent co-immunoprecipitations are shown. IP, immunoprecipitation. (**d**) Immunoblots showing H3K4me3, H3K4me1, histone H3 lysine 27 acetylation (H3K27ac), histone H3 lysine 27 trimethylation (H3K27me3) and total histone H3 in islets from *Dpy30*-WT and *Dpy30*-KO mice 45 days post tamoxifen administration. Numbers beneath each band indicate the band intensity normalised to the left-most sample. (**e**) Mean immunofluorescent intensity of DPY30, H3K4me3 and H3K4me1 in *Dpy30*-WT and *Dpy30*-KO beta cell nuclei at the indicated days after tamoxifen administration. Data are normalised to the fluorescence intensity of alpha cell nuclei (*n*=3). (**f**) Example immunohistochemical images of *Dpy30*-KO islets used for measurements in (**e**) showing H3K4me3 (cyan), insulin (magenta) and glucagon (yellow). Scale bars: 100 μm. (**g**, **h**) Scatterplots showing log_2_(fold change) and –log_10_(*p*) of gene expression in *Dpy30*-KO vs *Dpy30*-WT beta cells 15 days (**g**) and 45 days (**h**) post tamoxifen administration. Genes showing a twofold or greater increase or decrease in expression at *p*≤0.01 (calculated using Wald tests with Benjamini–Hochberg correction) are coloured red and green, respectively, and enumerated above. The full-length transcript of *Dpy30* is blue. (**i**, **j**) Blood glucose (**i**) and serum insulin (**j**) levels during an IPGTT in *Dpy30*-WT and *Dpy30*-KO mice 15, 30 and 45 days after tamoxifen administration. Data are means ± SD (*n*=8–15). *P* values were calculated from AUCs using multiple two-tailed *t* tests with Welch’s and Benjamini–Hochberg corrections. (**k**, **l**) Unfasted blood glucose levels (**k**) and body mass (**l**) of *Dpy30*-WT and *Dpy30*-KO mice up to 60 days after tamoxifen administration. Data are individual measurements with means (*n*=8; however, tracking was stopped after a blood glucose reading ≥20 mmol/l). *p<0.05, ***p<0.001

To induce synchronised deletion of *Dpy30* in mature beta cells, *Pdx1CreER*^*Tg/0*^;*Dpy30*^*flox/flox*^;*Rosa26*^*mTmG/+*^ mice were administered tamoxifen at 8 weeks of age (hereafter called *Dpy30*-KO mice) (Fig. 1a,b). As expected, *Dpy30*-KO did not impact assembly of the other core subunits of the COMPASS complex or their association with chromatin (Fig. 1c) but did reduce H3K4 methylation (Fig. 1d).

H3K4me3 and H3K4me1 were stable for at least 15 days after tamoxifen administration and were then gradually lost during the subsequent 45 days, whereas DPY30 was undetectable by 15 days (Fig. 1e,f). We reasoned that defects arising in *Dpy30*-KO beta cells by 15 days highlight functions of DPY30 itself. Defects arising later, by 45 days, highlight functions of H3K4 methylation. Remarkably, loss of DPY30 had little effect on gene expression in beta cells, with RNA-seq analysis identifying only three differentially expressed genes (*Dpy30*, *Edn3*, *C3*) 15 days post tamoxifen administration (Fig. 1g, ESM Tables 2 and 3). In contrast, 828 genes were dysregulated at 45 days, the majority of which (634) showed lower expression in *Dpy30*-KO cells (Fig. 1h, ESM Tables 4 and 5). We conclude that transcriptome remodelling in *Dpy30*-KO beta cells results from reduction of H3K4 methylation.

*Dpy30*-KO mice developed impaired glucose tolerance and had reduced serum insulin levels by 45 days post tamoxifen administration (Fig. 1i,j) and then rapidly developed diabetes (Fig. 1k,l), prompting euthanasia by 60 days. As the *Pdx1CreER* transgene may also drive recombination in delta cells [57] and the hypothalamus [58], we additionally used *Ins1*^*Cre*^ mice [46] to verify that *Dpy30* deletion specifically in beta cells is sufficient to drive the in vivo phenotype. Deletion of *Dpy30* from maturing beta cells using *Ins1*^*Cre*^ caused reduction of H3K4 methylation in islets by 5 weeks of age, leading to hyperglycaemia, impaired glucose tolerance and reduced serum insulin levels (ESM Fig. 1).

### H3K4 methylation maintains expression of genes involved in insulin production and glucose-stimulated activity

**Fig. 2:**
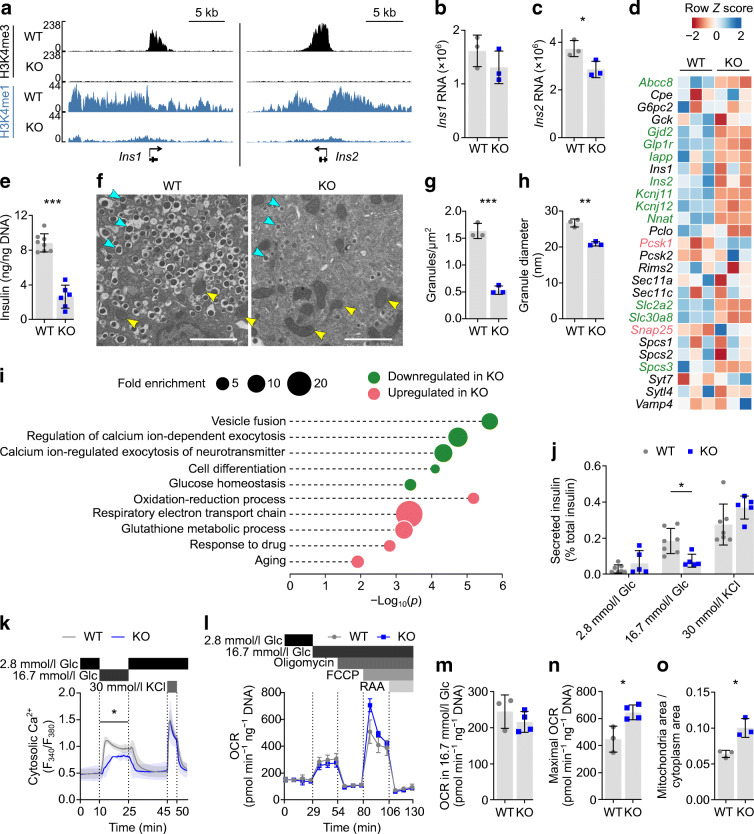
Genes involved in insulin production and glucose-induced activity are regulated by H3K4 methylation. (**a**) H3K4me3 and H3K4me1 enrichment at the *Ins1* and *Ins2* gene loci in *Dpy30*-WT and *Dpy30*-KO beta cells 45 days after tamoxifen administration. (**b**, **c**) *Ins1* (**b**) and *Ins2* (**c**) RNA levels in *Dpy30*-WT and *Dpy30*-KO beta cells 45 days after tamoxifen administration. Expression and *p* values were calculated from RNA-seq data using DESeq2 (Wald tests with Benjamini–Hochberg correction). (**d**) Heatmap showing expression *Z* scores in *Dpy30*-WT and *Dpy30*-KO beta cells 45 days post tamoxifen administration for selected genes. *P* values were calculated from RNA-seq data using Wald tests with Benjamini–Hochberg correction (*n*=3). Significantly downregulated and upregulated genes are shown in green and pink, respectively. (**e**) Insulin content in *Dpy30*-WT and *Dpy30*-KO islets. *P* values were calculated using two-tailed *t* tests with Welch’s correction (*n*=8 WT, *n*=6 KO). (**f**) Representative transmission electron micrographs of beta cells from a *Dpy30*-WT mouse and a *Dpy30*-KO mouse. Examples of insulin granules and mitochondria are indicated with cyan and yellow arrows, respectively. Scale bars: 2 μm. (**g**, **h**) Quantification of median insulin core granule density (**g**) and size (**h**). *P* values were calculated using two-tailed *t* tests with Welch’s correction (*n*=3). (**i**) Enrichment analysis of Gene Ontology: biological process terms in differentially expressed genes 45 days after tamoxifen administration. *P* values represent EASE scores, modified Fisher’s exact *p* values, calculated using the Database for Annotation, Visualization and Integrated Discovery (DAVID) v.6.8 [82]. (**j**) Insulin secretion from *Dpy30*-WT and *Dpy30*-KO islets during static in vitro stimulation with glucose and KCl solutions, normalised to islet insulin content. *P* values were calculated using multiple two-tailed *t* tests with Welch’s and Benjamini–Hochberg corrections (*n*=7 WT, *n*=5 KO). (**k**) Cytosolic Ca^2+^ concentration in islets from *Dpy30*-WT and *Dpy30*-KO mice during in vitro perifusion of glucose and KCl solutions. *P* values were calculated for the AUC in each time block by one-way ANOVA between genotypes (*n*=4 WT, *n*=3 KO). (**l**) Oxygen consumption rate in *Dpy30*-WT and *Dpy30*-KO dispersed islet cells during treatment with the indicated compounds (*n*=3 WT, *n*=4 KO). (**m**, **n**) Mitochondrial respiration in *Dpy30*-WT and *Dpy30*-KO islet cells in 16.7 mM glucose (**m**) and their maximal respiration capacity (**n**), inferred from the data shown in (**l**). *P* values were calculated using two-tailed *t* tests with Welch’s correction (*n*=3 WT, *n*=4 KO). (**o**) Fraction of cytoplasm area occupied by mitochondria in transmission electron micrographs of *Dpy30*-WT and *Dpy30*-KO beta cells. *P* values were calculated using two-tailed *t* tests with Welch’s correction (*n*=3). *p<0.05, **p<0.01, ***p<0.001. FCCP, carbonyl cyanide-p-trifluoromethoxyphenylhydrazone; Glc, glucose; OCR, oxygen consumption rate; RAA, rotenone and antimycin A

The insulin deficit in *Dpy30*-KO mice prompted us to examine insulin production and secretion. We first confirmed that H3K4me3 was lost from the *Ins1* and *Ins2* promoters in *Dpy30*-KO beta cells (Fig. 2a) 45 days after tamoxifen administration using ChIP-seq. H3K4me1 was also dramatically reduced (Fig. 2a). Despite this, RNA-seq data showed a surprisingly modest reduction in levels of *Ins1* and *Ins2* transcripts (Fig. 2b,c), indicating that robust expression of *Ins1* and *Ins2* can occur in the absence of local H3K4me3. However, insulin (but not glucagon) immunofluorescence intensity decreased in *Dpy3*0-KO mice (Fig. 1e and ESM Fig. 2a, b) and expression of several genes involved in insulin maturation and packaging was reduced (Fig. 2d, ESM Fig. 2c–g). This included *Nnat* (ESM Fig. 2d), encoding neuronatin, part of the signal peptidase complex and knockdown of which redirects preproinsulin for proteasomal degradation [59]. *Slc30a8*, encoding the zinc transporter ZNT8 required to package insulin into dense core granules, knockout of which impairs proinsulin-to-insulin processing [60], was also downregulated (ESM Fig. 2e). Consistent with impairment of insulin synthesis and/or increased degradation in *Dpy30*-KO mice, we observed a significant loss of islet insulin content (Fig. 2e). Reduction in insulin granule size and density in individual beta cells from *Dpy30*-KO mice was confirmed using electron microscopy (Fig. 2f–h). Therefore, *Dpy30*-KO in beta cells results in a reduction in insulin content.

In line with their lower insulin content, *Dpy30*-KO islets secreted less insulin during high glucose or KCl stimulation than *Dpy30* wild-type (WT) islets (ESM Fig. 2h). Gene Ontology analysis of downregulated genes suggested the additional impairment of biological processes required for stimulated insulin secretion, namely vesicle fusion, calcium-dependent exocytosis and glucose homeostasis (Fig. 2i, ESM Table 6). We tested whether this gene signature was associated with functional changes. When accounting for their low insulin content, *Dpy30*-KO islets responded normally to KCl, but glucose-stimulated insulin secretion remained low compared with *Dpy30*-WT islets (Fig. 2j). Cytosolic calcium influx was also impaired during high glucose but not KCl stimulation (Fig. 2k). Gene Ontology of upregulated genes prioritised energy production processes including oxidation/reduction and the electron transport chain (Fig. 2i, ESM Table 7); accordingly, mitochondrial respiratory capacity (Fig. 2l,n) and area (Fig. 2f,o) were elevated in *Dpy30*-KO cells. This was not associated with an elevated rate of glucose-stimulated respiration (Fig. 2m), perhaps because expression of the rate-limiting enzyme of glycolysis, *Gck*, was not altered (Fig. 2d, ESM Fig. 2f). These data suggest that *Dpy30*-KO impairs insulin secretion owing to a reduction of beta cell insulin content combined with a glucose-specific signalling defect downstream of mitochondrial glucose oxidation, although more work will be required to identify the precise mechanism. Together, these data show that genes with altered expression were enriched for roles in stimulation, secretion and energy production.

### Loss of H3K4 methylation is associated with limited gene downregulation in mature beta cells

**Fig. 3:**
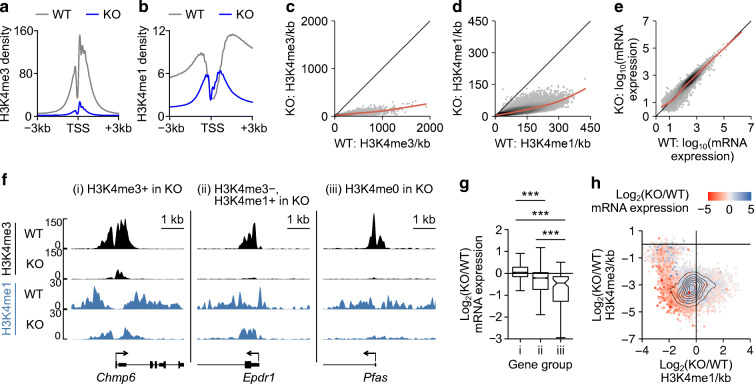
Transcription start site (TSS)-associated H3K4 methylation maintains gene expression in mature beta cells. (**a**, **b**) Average enrichment profiles of H3K4me3 (**a**) and H3K4me1 (**b**) at the TSS of all expressed genes in *Dpy30*-WT and *Dpy30*-KO chromatin. (**c**, **d**) Scatterplot of H3K4me3 (**c**) and H3K4me1 (**d**) enrichment in 10 kb bins spanning the genome in *Dpy30*-KO vs *Dpy30*-WT cells. (**e**) Scatterplot showing gene expression in *Dpy30*-KO vs *Dpy30*-WT cells. (**f**) Examples of genes that retain H3K4me3 (i), lose H3K4me3 but retain H3K4me1 (ii) and lose H3K4me3 and H3K4me1 (iii) from the TSS in *Dpy30*-KO chromatin. (**g**) Box and whisker plot showing log_2_(fold change) in RNA expression for gene groups exemplified in (**f**) (group i, *n*=10,045 genes, group ii, *n*=2302 genes, group iii, *n*=111 genes). (**h**) Contour scatterplot showing log_2_(fold change) in H3K4me1 enrichment (x axis) plotted against log_2_(fold change) in H3K4me3 enrichment (y axis) in the TSS±1 kb of all expressed genes. The log_2_(fold change) in RNA expression of the associated genes is shown as a colour gradient. ***p<0.001

While H3K4 methylation is predictive of transcription [31], our RNA-seq analysis indicates that only 634 of the 14,677 (4.3%) expressed genes were downregulated by depletion of H3K4 methylation (Fig. 1g, ESM Table 4). This suggests that H3K4 methylation is not required for the expression of most genes in mature beta cells. We therefore explored whether dysregulated genes were distinguished by specific epigenetic features. We first confirmed that H3K4 methylation was depleted at active promoters (Fig. 3a,b) and genome-wide (Fig. 3c,d) by *Dpy30*-KO. Despite this, the transcriptomes of *Dpy30*-KO and *Dpy30*-WT cells were well correlated and the total RNA expression of *Dpy30*-KO cells was not decreased (Fig. 3e). Therefore, global depletion of H3K4 methylation does not cause a global decrease in gene expression in mature beta cells.

To explore further, we segregated genes into three groups based on residual enrichment for H3K4 methylation, determined using model-based analysis of ChIP-seq (MACS2) [61]. Group (i) retained at least some H3K4me3 enrichment at the transcription start site (TSS) (10,045 genes); group (ii) lost H3K4me3 but retained at least some H3K4me1 enrichment (2302 genes); and group (iii) lost H3K4me3 and H3K4me1 (111 genes) (Fig. 3f, ESM Table 8). As a group, genes that retained at least some enrichment for H3K4me3 at the TSS were not downregulated in *Dpy30*-KO cells (Fig. 3g). Loss of both H3K4me3 and H3K4me1 from the TSS was associated with significant impairment of gene expression, and genes that lost H3K4me3 but retained H3K4me1 showed an intermediate effect (Fig. 3g). To visualise this relationship in a threshold-free manner, we plotted the change in H3K4me3, H3K4me1 and RNA expression for all active TSSs (Fig. 3h). Most genes clustered in the bottom left quadrant, indicating some reduction of both H3K4me3 and H3K4me1 from the TSS. Strikingly, genes that were highly downregulated clustered at the extreme edge, indicating that genes experiencing the greatest relative loss of both H3K4me3 and H3K4me1 were strongly downregulated (Fig. 3h). Thus, while transcriptome remodelling in this model is likely to reflect both direct and indirect effects of reduced methylation, these data show that loss of H3K4me3 from a gene promoter was associated with reduced expression of that gene.

### H3K4 methylation enforces expression of weakly active and repressed genes in mature beta cells

**Fig. 4:**
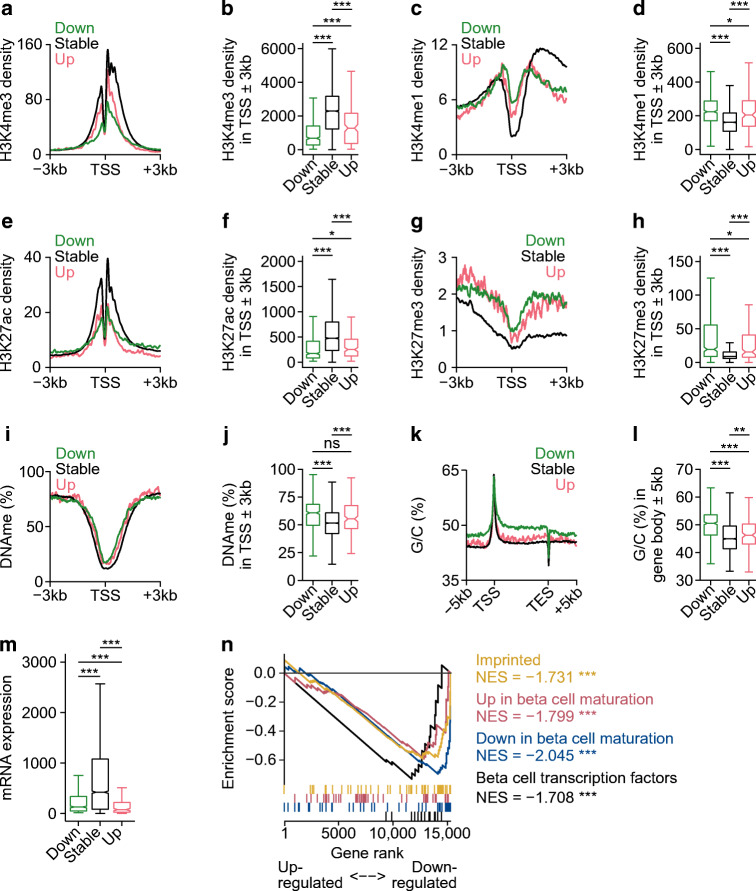
Weakly active and developmentally regulated genes are susceptible to downregulation in H3K4me3-deficient beta cells. (**a**–**l**) Average enrichment profiles and quantification of H3K4me3 (**a**, **b**), H3K4me1 (**c**, **d**), H3K27ac (**e**, **f**), H3K27me3 (**g**, **h**), DNAme (**i**, **j**) and G/C nucleotide content (**k**, **l**) in *Dpy30*-WT cells for genes downregulated, stably expressed or upregulated by *Dpy30*-KO. (**m**) Box and whisker plot showing RNA expression in *Dpy30*-WT cells for genes downregulated, stably expressed or upregulated by *Dpy30*-KO. (**n**) Running enrichment plot for imprinted genes ([83]), genes up- or downregulated during mouse beta cell maturation (adults vs postnatal day 10, [63]) and mature beta cell transcription factor genes. *P* values were calculated using Wilcoxon rank-sum (**b**–**m**) or permutation (**n**) tests with Benjamini–Hochberg correction. *p<0.05, **p<0.01, ***p<0.001. NES, normalised enrichment score; ns, not significant; TES, transcription end site

We compared the chromatin profile of downregulated and stably expressed genes by measuring histone H3 lysine 27 trimethylation (H3K27me3), a marker of developmentally repressed chromatin, and histone H3 lysine 27 acetylation (H3K27ac), a marker of active chromatin, using ChIP-seq. We also retrieved data for DNA cytosine methylation (DNAme), a modification linked to gene silencing, in C57BL6 mouse beta cells [55], and examined nucleotide content. The downregulated gene set had a weakly active profile even in *Dpy30*-WT cells: enrichment for H3K4me3 and H3K27ac was low whereas H3K4me1, H3K27me3, G/C content, and DNAme were high in comparison to stably expressed genes (Fig. 4a–l). Accordingly, the downregulated gene set showed lower expression in *Dpy30-*WT cells than stably expressed genes (Fig. 4m). Interestingly, genes upregulated in *Dpy30*-KO cells had a similar epigenetic profile to downregulated genes (Fig. 4a–m).

### H3K4 methylation deficiency leads to a less active and more repressed epigenome in mature beta cells

**Fig. 5:**
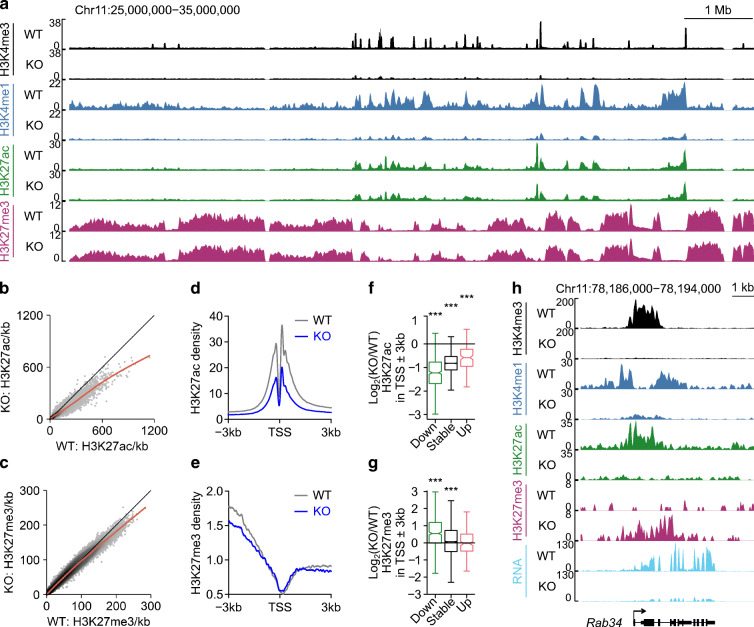
H3K4me3 deficiency leads to a less active and more repressed epigenome in mature beta cells. (**a**) Genome browser view of H3K4me3, H3K4me1, H3K27ac and H3K27me3 in *Dpy30*-WT and *Dpy30*-KO cells showing preservation of the megabase-scale organisation of H3K27me3-positive Polycomb-repressed compartments. (**b**, **c**) Scatterplots of H3K27ac (**b**) and H3K27me3 (**c**) enrichment in 10 kb bins spanning the genome in *Dpy30*-KO vs *Dpy30*-WT cells. (**d**, **e**) Average enrichment profiles of H3K27ac (**d**) and H3K27me3 (**e**) at the TSS of all expressed genes in *Dpy30*-WT and *Dpy30*-KO cells. (**f**, **g**) Box and whisker plots showing the log_2_(fold change) in H3K27ac (**f**) and H3K27me3 (**g**) enrichment in the TSS of downregulated, stable and upregulated genes in *Dpy30*-KO vs *Dpy30*-WT cells. (**h**) Genome browser view of a downregulated gene, *Rab34*, showing loss of H3K4me3, H3K4me1, H3K27ac and RNA and accumulation of H3K27me3 in *Dpy30*-KO cells. *P* values were calculated using Wilcoxon rank-sum tests with Benjamini–Hochberg correction. ***p<0.001

During development, active (H3K27ac-positive) and repressed (H3K27me3- or DNAme-positive) chromatin is dynamically regulated; resolution of the genome into active and repressed domains is fundamental to establishment of terminal cell type-specific transcriptomes [29, 62]. We noted that expression of genes that are dynamically regulated during, and important for, beta cell maturation [63] tended to be lower in *Dpy30*-KO cells (Fig. 4n), suggesting that maintenance of genes in an active state requires continual reinforcement in mature beta cells. Because G/C content tended to be high at downregulated gene loci (Fig. 4k,l) and imprinted genes tended to be downregulated in *Dpy30*-KO cells (Fig. 4n), we measured DNAme near the TSS of three downregulated genes (*Cdkn1c*, *Igf2* and *Cd81*) but did not observe a change (ESM Fig. 3), suggesting that reduction of H3K4me3 did not immediately lead to accumulation of DNAme. We explored whether H3K27me3-positive regions expand in H3K4me3-deficient cells. As shown in Fig. 5a, the megabase-scale distribution of H3K27me3-positive compartments was largely unchanged in H3K4me3-deficient beta cells. However, H3K27me3 increased specifically at promoters of downregulated genes (Fig. 5g,h). These data suggest that maintenance of H3K4 trimethylation is necessary to prevent Polycomb-mediated gene repression in mature beta cells.

In contrast to the locus-specific accumulation of H3K27me3, H3K27ac was diminished throughout the genome in *Dpy30*-KO cells (Fig. 5b). Like H3K4me, H3K27ac was reduced even at promoters of genes that were stably expressed or upregulated in *Dpy30*-KO cells (Fig. 5d,f), indicating that the generalised decrease in H3K27ac was not a consequence of gene downregulation. Despite the global reduction, however, H3K27ac was still positively associated with gene expression in *Dpy30*-KO chromatin: H3K27ac peaks gaining intensity were near upregulated genes, and H3K27ac peaks losing intensity were near downregulated genes, more frequently than expected by chance (ESM Fig. 4). These observations are consistent with a model wherein acetylation of H3K27 is partially a consequence of H3K4 methylation [18] and of transcription [64].

To summarise, *Dpy30*-KO beta cells showed a global reduction in active histone marks but not of gene expression. Genes that lost an especially large fraction of H3K4 methylation tended to be downregulated; they tended to be developmentally regulated or in a weakly active state in *Dpy30*-WT beta cells and to accumulate the repressive mark H3K27me3, which was otherwise globally stable.

### Reduction in transcriptional consistency in *Dpy30*-KO beta cells

**Fig. 6:**
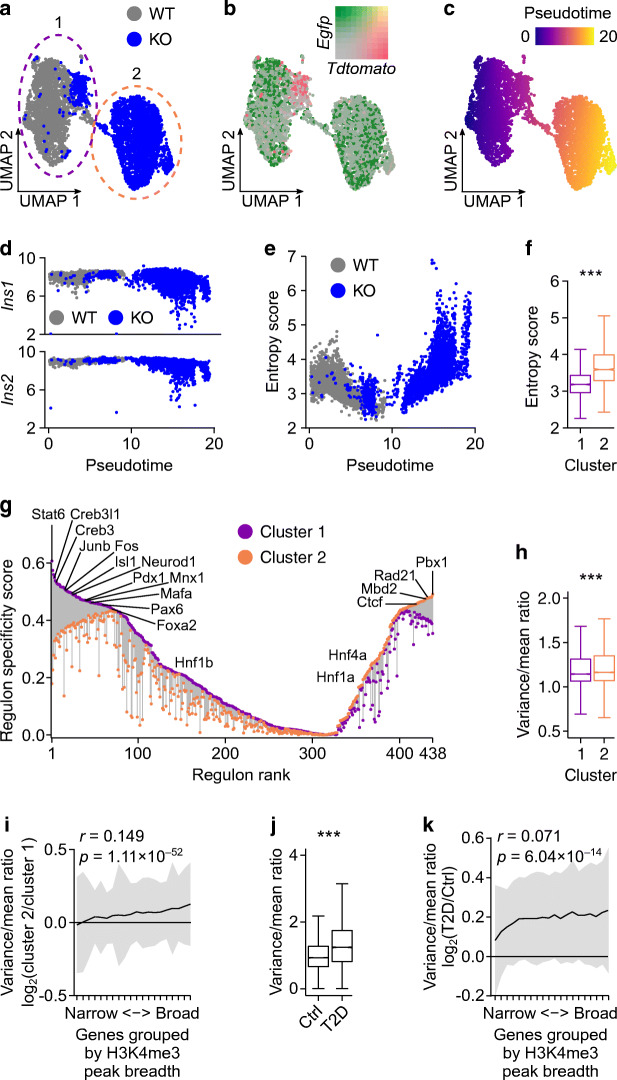
Reduction in transcriptional consistency in beta cells from *Dpy30*-KO mice and human donors with type 2 diabetes. (**a**–**c**) Uniform manifold approximation and projection (UMAP) visualisation of beta cell transcriptomes coloured according to genotype of the donor mouse (**a**), expression of *tdTomato*, *EGFP* and their overlap (**b**) and pseudotime (**c**). (**d**) Scatterplot showing *Ins1* and *Ins2* expression in beta cells plotted against pseudotime. (**e**) Scatterplot showing gene expression entropy scores of beta cells plotted against pseudotime. (**f**) Box and whisker plot showing transcriptome entropy of beta cells in clusters 1 and 2 (2466 and 2411 cells, respectively). (**g**) Active transcription factor regulons identified using SCENIC [67, 68], showing the specificity score of each regulon for cluster 1 (purple) and cluster 2 (orange) connected by a line. Regulons are ranked from the most specific for cluster 1 on the left to the most specific for cluster 2 on the right, and some notable regulons are labelled. (**h**) Box and whisker plot showing the variance/mean ratio of gene expression across beta cells in clusters 1 and 2. (**i**) Variance/mean ratio of beta cell gene expression in cluster 2 vs cluster 1 (y axis) plotted as a function of H3K4me3 breadth in quantiles (x axis) (622 genes/quantile). Data are presented as means ± SD. The Spearman rank correlation is shown. (**j**, **k**) The same analysis as in (**h**) and (**i**), respectively, in beta cells from human donors with or without type 2 diabetes, using H3K4me3 ChIP-seq data from Bramswig et al [52] and single-cell RNA-seq (scRNA-seq) data from Camunas-Soler et al [54]. *P* values were calculated using Wilcoxon rank-sum tests. ***p<0.001

Transcriptional entropy describes the ‘specialisation’ of a cell’s transcriptome whereby cells with low entropy have a narrow distribution of highly expressed genes and gene expression in cells with high entropy becomes less predictable. In islets, transcriptional entropy decreases during maturation [65] and increases in type 2 diabetes [4]. We explored whether reduction of H3K4 methylation increases the variability or entropy of gene expression. To this end, we processed islets from a *Dpy30*-WT mouse and a *Dpy30*-KO mouse for single-cell RNA-seq (ESM Fig. 5a). We obtained transcriptome profiles of 4877 beta cells, which formed two distinct clusters: cluster 1 contained cells from the *Dpy30*-WT mouse and a small population of beta cells from the *Dpy30*-KO mouse expressing *tdTomato* (indicating that they were not Cre-recombined) and cluster 2 contained *EGFP*-expressing *Dpy30-*KO beta cells (Fig. 6a,b). To model the divergence of *Dpy30*-KO beta cells from *Dpy30*-WT and unrecombined beta cells, we performed pseudotime trajectory analysis using Slingshot [66] (Fig. 6c). Expression of *Ins1* and *Ins2* showed a narrow distribution in *Dpy30*-WT and unrecombined beta cells but became more variable as pseudotime progressed in *Dpy30*-KO cells (Fig. 6d). More generally, overall transcriptional entropy increased in *Dpy30*-KO cells over pseudotime (Fig. 6e) and tended to be higher than in *Dpy30*-WT and unrecombined beta cells (Fig. 6f). As lower entropy is associated with greater cell maturity [65], we examined the expression of genes associated with immaturity and dedifferentiation but did not observe induction of these genes in *Dpy30*-KO cells (see ESM Fig. 5b, c and compare with Fig. 4n). Therefore, *Dpy30*-KO led to higher transcriptional entropy in mature beta cells but did not cause reversion to a developmentally immature transcriptional state.

A rise in entropy implies a loss of transcriptional control. To identify transcription factors whose activity is altered following loss of *Dpy30* and H3K4 methylation in mature beta cells we used single-cell regulatory network inference and clustering (SCENIC [67, 68]). Unsupervised clustering of beta cells on the basis of their regulon activities (i.e. expression of transcription factors and their putative targets) effectively separated *Dpy30*-KO beta cells from *Dpy30*-WT and unrecombined beta cells (ESM Fig. 6a, b), recreating clusters based on gene expression profiles (ESM Fig. 6c) and indicating a shift in the gene regulatory landscape after *Dpy30*-KO. Of 438 active regulons identified in beta cells, 319 (73%) showed lower activity in *Dpy30*-KO cells (cluster 2) (Fig. 6g, ESM Table 9). In line with the general downregulation of mature beta cell transcription factor genes (Fig. 4n), Foxa2, Isl1, Mafa, Mnx1, Neurod1, Pax6 and Pdx1 showed lower activity in *Dpy30*-KO cells cluster 2) (Fig. 6g, ESM Fig. 6d). Activity-regulated factors such as Fos and Junb, and Creb3 factors, which regulate secretory pathways [69], also showed lower activity (Fig. 6g, ESM Fig. 6d). Regulons showing greater activity in the *Dpy30-*KO cluster 2 included Ctcf and Rad21, which independently regulate chromatin organisation [70], and Mbd2, which binds to a methylated DNA motif [71] (Fig. 6g, ESM Fig. 6d, ESM Table 9). This analysis suggests that coordination between chromatin structure and transcription factor activity is altered in *Dpy30*-KO cells.

We examined whether transcriptional consistency, that is, the variability of gene expression between cells, was linked to the breadth of the H3K4me3 peak at gene promoters, as broad H3K4me3 peaks may increase transcriptional consistency [27]. We ranked H3K4me3 peaks in *Dpy30*-WT cells in order of increasing breadth and grouped them into 20 quantiles, each representing 5% of the H3K4me3 peaks (ESM Table 10). We then compared the variance in expression of genes with H3K4me3 peaks in each quantile between beta cell clusters. Transcriptional variability between cells was increased in *Dpy30*-KO cells (Fig. 6h). Greater H3K4me3 peak breadth was modestly correlated with a greater gain of variability in *Dpy30*-KO cells (Fig. 6i; Pearson *r*=0.149, *p*=1.11 × 10^−52^). Analysis of H3K4me3 ChIP-seq data from healthy human beta cells [52] and single-cell RNA-seq data from human donors with or without type 2 diabetes [54] suggests that variability of gene expression is also higher in type 2 diabetes (Fig. 6j) and that the gain in variability is weakly correlated with H3K4me3 peak breadth (Fig. 6k; Pearson *r*=0.071, *p*=6.04 × 10^−14^). These data support a minor association between broad H3K4me3 peaks and transcriptional consistency in beta cells, which is nominally linked to the increased transcriptional variability in type 2 diabetes.

### H3K4me3 peak breadth stratifies genes dysregulated in type 2 diabetes

**Fig. 7:**
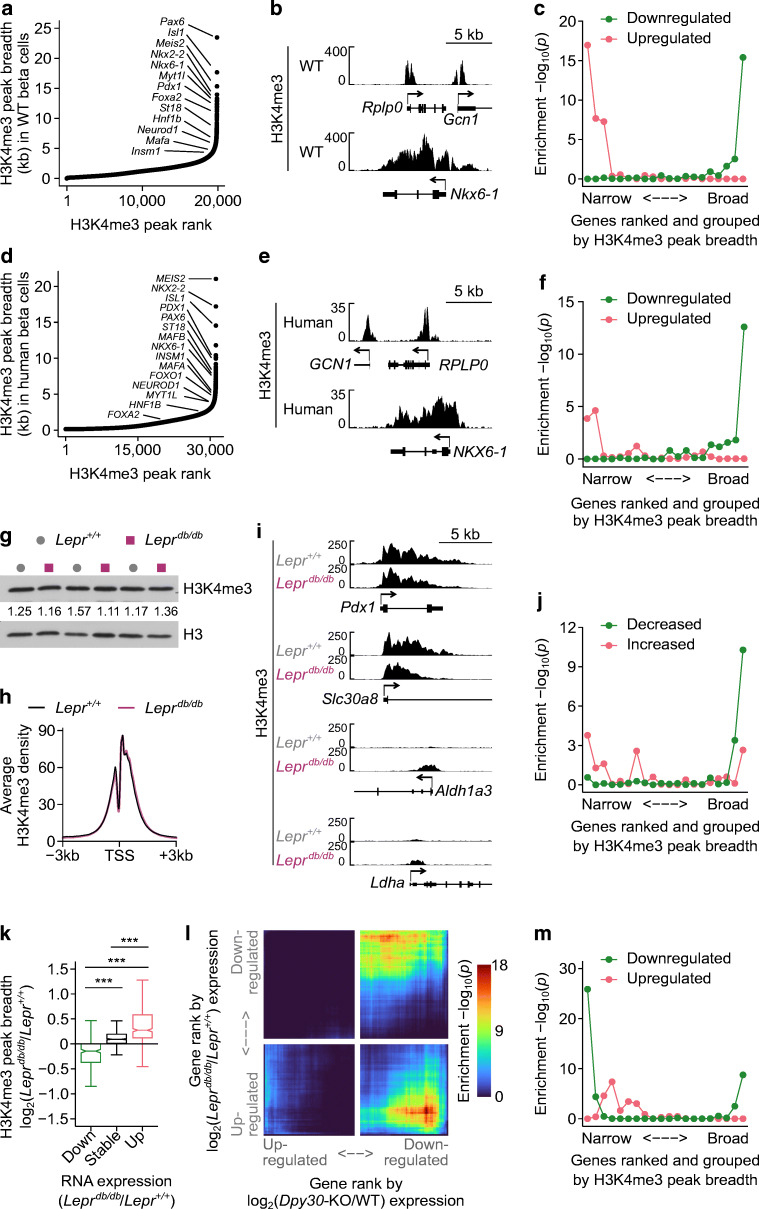
H3K4me3 peak breadth encodes gene expression changes in a mouse model of type 2 diabetes. (**a**) H3K4me3 peaks in *Dpy30*-WT beta cells ranked from narrow to broad. Peaks associated with beta cell transcription factor genes are labelled. (**b**) Genome browser views showing H3K4me3 at a housekeeping gene (*Rplp0*) and an expression-matched beta cell transcription factor gene (*Nkx6-1*). (**c**) Enrichment *p* values of genes down- or upregulated in islets from *Lepr*^*db/db*^ mice, with genes ranked by H3K4me3 peak breadth (as in [**a**]) and grouped into 20 quantiles. *P* values were calculated using one-sided Fisher’s exact tests. (**d**, **e**) The same data as in (**a**, **b**) for human beta cells. (**f**) Enrichment *p* values of genes down- or upregulated in islets from donors with type 2 diabetes, with genes ranked by H3K4me3 peak breadth (as in [**d**]) and grouped into 20 quantiles. *P* values were calculated using one-sided Fisher’s exact tests. (**g**) Immunoblots of H3K4me3 and H3 in islet lysates from *Lepr*^*+/+*^ and *Lepr*^*db/db*^ mice. H3-normalised H3K4me3 band densities are listed. (**h**) Average enrichment profiles of H3K4me3 at the TSS of all expressed genes in *Lepr*^*+/+*^ and *Lepr*^*db/db*^ islets. (**i**) Genome browser views of H3K4me3 in *Lepr*^*+/+*^ and *Lepr*^*db/db*^ islets at notable genes that are downregulated (*Pdx1*, *Slc30a8*) or induced (*Aldh1a3*, *Ldha*) in *Lepr*^*db/db*^ islets. (**j**) Enrichment *p* values of H3K4me3 peaks showing significant change in peak breadth in *Lepr*^*db/db*^ vs *Lepr*^*+/+*^ islets (y axis) in the different gene groups ranked from narrow to broad (x axis). Enrichment *p* values were calculated using one-sided Fisher’s exact tests. (**k**) Box and whisker plot showing the log_2_(fold change) in H3K4me3 peak breadth for genes that are downregulated, stably expressed and upregulated in *Lepr*^*db/db*^ islets. *P* values were calculated using Wilcoxon rank-sum tests with Benjamini–Hochberg correction. ***p<0.001. (**l**) Stratified rank/rank hypergeometric overlap plot comparing gene expression changes caused by *Dpy30*-KO (x axis) and by *Lepr*^*db/db*^ (y axis) mutations. Colourscale shows the hypergeometric enrichment *p* value. (**m**) Same as (**c**) for genes down- or upregulated in *Dpy30*-KO cells

We explored the relationship between H3K4me3 peak breadth, gene expression and diabetes further. H3K4me3 forms 18,936 distinct peaks in beta cells. Ranking peaks by breadth revealed a class of exceptionally broad peaks spanning up to 21 kb, including those at essential beta cell transcription factor genes (Fig. 7a, ESM Table 10), consistent with the observation that genes marked by broad peaks are important for maintaining cell identity [27]. For example, Fig. 7b shows the H3K4me3 profiles of a housekeeping gene with a typical H3K4me3 peak (*Rplp0*), and an expression-matched beta cell transcription factor gene with a broad peak (*Nkx6-1*). Gene expression was moderately correlated with promoter H3K4me3 peak breadth; however, genes with exceptionally broad peaks did not have exceptionally high levels of RNA expression (ESM Fig. 7a).

To explore the relationship between H3K4me3 peak breadth and diabetes, we used the *Lepr*^*db/db*^ mouse model of type 2 diabetes. We retrieved published bulk RNA-seq data from *Lepr*^*db/db*^ islets [53] and compared H3K4me3 peak breadth with gene expression changes. Notably, genes that become downregulated in *Lepr*^*db/db*^ islets were explicitly enriched in the group of genes with broad H3K4me3 peaks (Fig. 7c). Furthermore, housekeeping genes (GSEA:M11197 [72]), genes involved in endocrine pancreas development (GSEA:M12875) and MODY genes (GSEA:M18312) were enriched in the broadest H3K4me3 peaks (ESM Fig. 7b). In contrast, genes upregulated in *Lepr*^*db/db*^ islets were uniquely enriched in the narrowest H3K4me3 peaks (Fig. 7c). As H3K4me3 peak breadth correlates with gene expression (ESM Fig. 7a), we tested whether these observations could be secondary to a relationship wherein highly expressed genes become downregulated and weakly expressed genes become upregulated in *Lepr*^*db/db*^. Ranking genes by RNA expression level instead of H3K4me3 peak breadth proved less effective at stratifying each gene set except for housekeeping genes (ESM Fig. 7b), supporting a primary link with H3K4me3. Therefore, genes that are differentially expressed in diabetic islets are diametrically stratified according to H3K4me3 breadth, with upregulated genes having narrow peaks and downregulated genes having broad peaks in non-diabetic conditions. These observations are replicable in humans using publicly available H3K4me3 ChIP-seq data from beta cells from donors without diabetes [52] and RNA-seq data from islets from donors with or without type 2 diabetes [51] (Fig. 7d–f, ESM Fig. 7c, d, ESM Table 11).

### H3K4me3 peak breadth dynamics encode gene expression changes in a mouse model of type 2 diabetes

The positive association between H3K4me3 peak breadth and differential gene expression in diabetes prompted us to examine if H3K4me3 is altered in diabetic islets. Several COMPASS subunit genes were downregulated in *Lepr*^*db/db*^ islets (ESM Fig. 8a), although none reached statistical significance in islets from human donors with diabetes (ESM Fig. 8b). Immunoblots and ChIP-seq showed that average H3K4me3 enrichment was not altered in *Lepr*^*db/db*^ islets (Fig. 7g,h). However, we observed contraction of H3K4me3 at downregulated genes, for example the transcription factor *Pdx1* and zinc transporter *Slc30a8*, and expansion of H3K4me3 at upregulated genes, for example the disallowed *Aldh1a3* and *Ldha* (Fig. 7i). RNA expression and H3K4me3 peak breadth decreased for mature beta cell transcription factor genes and increased for disallowed genes (ESM Fig. 9a, b). More generally, broad H3K4me3 peaks tended to shrink and narrow H3K4me3 peaks tended to expand in *Lepr*^*db/db*^ islets (Fig. 7j). Similarly, genes that were up- or downregulated in *Lepr*^*db/db*^ islets showed corresponding expansion or contraction of HK4me3 levels, respectively (Fig. 7k). Therefore, a pattern emerges in *Lepr*^*db/db*^ islets wherein weakly active genes tend to gain H3K4me3 and become upregulated, while genes with broad H3K4me3 peaks tend to lose some H3K4me3 and become downregulated.

We examined whether genes that are dysregulated in *Lepr*^*db/db*^ islets are regulated by H3K4 methylation, that is, are downregulated in *Dpy30*-KO cells 45 days after tamoxifen administration. To test the overlap between differential gene expression in the *Dpy30*-KO and *Lepr*^*db/db*^ models, we used a threshold-free rank–rank hypergeometric overlap test [73]. There was significant overlap between genes downregulated in *Dpy30*-KO mice with genes that were either up- or downregulated in *Lepr*^*db/db*^ mice (Fig. 7l). Accordingly, genes downregulated in *Dpy30*-KO mice were enriched in both the narrow and broad H3K4me3 peak groups (Fig. 7m), thus mirroring the pattern for genes up- or downregulated in *Lepr*^*db/db*^ mice (Fig. 7c). Gene Ontology terms for insulin secretion, response to glucose and regulation of transcription were over-represented in genes downregulated in both models (ESM Fig. 9c, ESM Tables 12 and 13). Terms over-represented in genes downregulated in *Dpy30*-KO but upregulated in *Lepr*^*db/db*^ included regulation of cell growth, transport and response to unfolded protein and endoplasmic reticulum stress (ESM Fig. 9d, ESM Table 14, 15), which could indicate that *Dpy30*-KO mice are not subject to these stresses and/or that methylation of H3K4 is required for induction of stress-responsive genes, as previously suggested in *Drosophila* [20]. Combined, these results suggest that genes that are dysregulated in *Lepr*^*db/db*^ islets show concordant changes in H3K4me3 peak breadth and significantly overlap with genes that are downregulated in *Dpy30*-KO cells, which perform contextually important biological functions in mature beta cells.

## Discussion

**Fig. 8:**
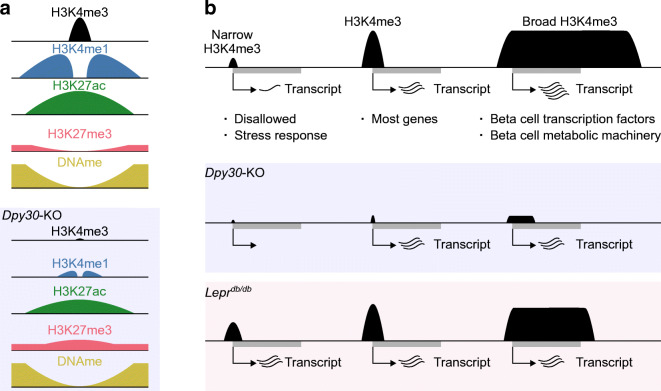
Summary of H3K4me3 in mature mouse beta cells. (**a**) Active gene promoters are enriched for H3K4me3, H3K4me1 and H3K27ac and are depleted of repressive H3K27me3 and DNAme. Reduction of H3K4me3 and H3K4me1 in *Dpy30*-KO cells was associated with a generalised reduction of H3K27ac and specific gain of H3K27me3 in downregulated gene promoters. (**b**) A continuum of H3K4me3 peak breadth distinguishes disallowed and lowly expressed genes from critical mature beta cell lineage factors. Global reduction of H3K4me3 in *Dpy30*-KO cells impaired expression of genes with very narrow or very broad peaks. In *Lepr*^*db/db*^ mice, accumulation of H3K4me3 at narrow peaks and loss of H3K4me3 from broad peaks was associated with concordant changes in gene expression. Genes with an intermediate H3K4me3 peak profile were not generally dysregulated in either model

In this study we describe a gene regulatory system in mature beta cells with consequences for cell function in health and type 2 diabetes. We find that sustained methylation of H3K4 is essential for the preservation of insulin content and stimulated secretion. Reduction of H3K4 methylation led to a less active and more repressed epigenome. Gene expression became more stochastic, a feature shared with beta cells during type 2 diabetes. We furthermore describe epigenetic features associated with genes that are sensitive to H3K4 demethylation in beta cells in vivo, providing support for a generalisable, but limited, function for H3K4me3 in the regulation of transcription in a terminally differentiated tissue in vivo (Fig. 8).

Conditional mutagenesis of *Dpy30* revealed a slow turnover of H3K4 methylation in mature beta cells. We observed slower loss of H3K4me1 than H3K4me3, consistent with previously reported relative turnover rates [74], and that loss of methylation was probably driven by enzymatic demethylation and nucleosome eviction rather than dilution due to mitosis or overall H3 degradation [74, 75]. Following an initial delay of between 15 and 30 days after tamoxifen administration, H3K4 methylation was progressively lost from beta cells, leading to impaired glucose tolerance by 45 days, and hyperglycaemia shortly thereafter. The initial delay may result from the action of pre-existing DPY30, while the metabolic dysfunction appearing by 45 days apparently represents a threshold beyond which methylation was too low to sustain normal beta cell functions. Then, unabated decline of H3K4 methylation and/or a feed-forward of beta cell dysfunction led to worsening hyperglycaemia and euthanasia by 60 days. Although reduction of methylation was obvious by 30 days after tamoxifen administration, no defect in glucose tolerance was observed at this time point, indicating that beta cells can maintain normal function when faced with a moderate reduction in methylation levels. This suggests that therapeutic strategies that cause limited demethylation of H3K4 could be tolerated, although long-term effects were not examined here. Further, this may explain why human pathologies caused by heterozygous mutations in COMPASS methyltransferases, such as Wiedemann–Steiner syndrome (caused by mutations in *KMT2A*) and subgroups of generalised dystonia (*KMT2B*) and Kabuki syndrome (*KMT2D*), do not present with glucose intolerance and hyperglycaemia; compensation by other methyltransferases means that it is unlikely that H3K4me3 is reduced to an extent that impairs beta cell function.

DPY30 was depleted in *Dpy30*-KO cells by 15 days after tamoxifen administration but no phenotype was apparent until H3K4me3 was also depleted, several weeks later. This suggests that DPY30 has little direct impact on gene expression or insulin secretion by beta cells. Still, we cannot exclude the possibility that DPY30 stimulates methylation of stable non-histone substrates, or contributes to non-transcriptional functions, with a subtle or delayed phenotype. For instance, SETD1B-COMPASS has a non-enzymatic role in cytosolic lipid metabolism [36]. Lipid metabolism potentiates glucose-stimulated insulin secretion [76] so, if DPY30 contributes to this role, its ablation conceivably contributed to the impaired glucose-stimulated insulin secretion we observed.

In *Dpy30*-KO beta cells, changes in RNA expression were linked to local changes in histone methylation, leading to dysregulation of ~5% of genes. This mirrors findings in yeast [25], *Drosophila* embryos [19] and mouse embryonic stem cells [18] that H3K4 methylation is not required to maintain expression of most genes. H3K4 methylation may, therefore, be important for allocation of transcriptional machinery to particular genes, rather than their absolute activity. In other words, promoter-associated H3K4me3 levels modulate expression of target genes but global H3K4me3 levels should not be assumed to reflect a cell’s transcriptional output. We note, however, that total mature mRNA content, which we measured here, is not a direct measure of transcription, and measurement of nascent RNA may have uncovered more widespread roles in transcription regulation. In either case, our findings raise the important question of why reduction of H3K4me3 was associated with downregulation of a specific group of H3K4me3-marked genes. Part of the answer probably arises from cooperation with specific transcription factors. For example, there is evidence that MAF bZIP transcription factor A (MAFA) and paired box 6 (PAX6) activate target genes by recruiting COMPASS methyltransferases [77, 78] and the activity of both was decreased in *Dpy30*-KO cells. Additional specificity probably arises from compensation and competition from other epigenetic regulators. H3K27ac, an unambiguous marker of active chromatin, was globally reduced in *Dpy30*-KO cells and was positively linked to gene up- and downregulation. H3K27me3, a modification that antagonistically drives gene repression, accumulated at the promoters of downregulated genes. Thus, our results are consistent with a cooperative role for H3K4me3 within a larger ‘histone code’ [79]. We focused our analyses on promoters but it should be noted that H3K4 methylation also influences gene expression from distal enhancers [15]. We suspect that consideration of enhancers would further refine the link between gene regulation and H3K4 methylation in beta cells. More broadly, we believe that, provided one has sufficient knowledge of the epigenetic profile of a given gene, the degree to which its expression depends on H3K4 methylation could be accurately predicted.

Global reduction of H3K4 methylation was associated with upregulation of almost 200 genes. This observation is at odds with correlative and mechanistic evidence that H3K4 methylation is an ‘activating’ mark [13, 14, 26] but it is a common observation when COMPASS genes are inactivated (e.g. [20, 24, 25, 43]). Although the mechanism by which this occurs is unknown, it may point to roles for COMPASS/H3K4 methylation in gene repression. In this regard, it is notable that epigenetic features were similar between upregulated and downregulated genes (Fig. 4). It is tempting to speculate that, in the same way that transcription factors can activate or repress target genes from identical DNA motifs, COMPASS/H3K4 methylation can activate or repress genes with common epigenetic features based on the influence of other regulatory inputs. Alternatively, reduction of H3K4 methylation is merely permissive of gene upregulation caused by other factors, such as (1) downregulation of negative transcriptional regulators, (2) metabolic stimuli and (3) reallocation of transcriptional machinery from downregulated genes. Whether H3K4me3 directly suppresses expression in some circumstances will need to be tested by carrying out acute targeted methylation and demethylation at candidate genes.

Peak breadth has emerged as an informative dimension of H3K4me3 enrichment: H3K4me3 peak breadth predicts gene expression levels [31]; breadth dynamics predict gene expression dynamics [80]; and exceptionally broad H3K4me3 peaks are predictive of genes that are critical to the maintenance of a cell’s lineage [27]. From our analyses of peak breadth, we confirmed that markers of beta cells, including *Ins1*/*2* and beta cell transcription factors, possess broad H3K4me3 peaks in mature beta cells. *Dpy30*-KO led to a genome-wide reduction of H3K4me3 but transcriptional downregulation was largely restricted to genes that had either very narrow or very broad H3K4me3 peaks, indicating that these two classes of promoters rely on H3K4me3 for the maintenance of gene expression. Compared with the effect at narrow peaks, the transcriptional defects for genes with broad H3K4me3 enrichment was modest. For example, *Ins1* and *Ins2* were downregulated by 15–25% in *Dpy30*-KO cells despite complete loss of their broad H3K4me3 peaks. As broad peaks colocalise with many other activating chromatin marks [27], the modest effect is likely to result from compensation from other regulatory inputs. In aggregate, our results show that broad H3K4me3 peaks increase expression levels and transcriptional consistency of associated genes but are not required for transcription.

A surprising finding is that weakly active promoters, with little H3K4me3, show the greatest reliance on H3K4 methylation. One explanation is that these promoters are susceptible to silencing as H3K4 methylation opposes repressive H3K27 and DNA methyltransferase activity [16, 17]. In support of this, genes that were downregulated in *Dpy30*-KO cells showed high initial enrichment of H3K27me3 and further accumulation after reduction of H3K4me3. Weakly active genes are also the most likely to be upregulated after inactivation of the Polycomb system [81] and, in beta cells, inactivation of the Polycomb system leads to activation of previously silenced genes [4]. Therefore, weakly active genes may be the most responsive targets in ongoing competition between the Polycomb and trithorax systems in mature beta cells.

Weakly active genes become more active in *Lepr*^*db/db*^ islets, suggesting that the trithorax system is dominant during the development of diabetes. Meanwhile, promoters with broad H3K4me3 peaks showed a unique tendency to shrink and for the associated gene to be downregulated. More work is required to determine what drives H3K4me3 peak dynamics in *Lepr*^*db/db*^ islets and to what extent these mechanisms are active in human diabetes. In any case, we favour a model wherein redistribution of H3K4me3 away from promoters of genes with broad H3K4me3 peaks, which enforce beta cell identity or functions, in favour of relatively inactive genes, including stress response and disallowed genes, contributes to transcriptome remodelling and type 2 diabetes.

## Supplementary information

ESM (PDF 4873 kb)

ESM Tables (XLSX 8344 kb)

## Data Availability

RNA-seq, scRNA-seq and ChIP-seq data generated from *Pdx1CreER Dpy30*-KO islet cells and ChIP-seq data generated in *Lepr*^*db/db*^ islet cells have been deposited in the Gene Expression Omnibus under accession number GSE181951. Requests for further information should be directed to the corresponding author, Brad G. Hoffman (brad.hoffman@ubc.ca).

## Abbreviations

ASH2L: Absent, small or homeotic 2-like protein
ChIP-seq: Chromatin immunoprecipitation–sequencing
COMPASS: Complex of proteins associated with Set1
DNAme: DNA cytosine methylation
DPY30: Dumpy-30
*Dpy30*-KO: Beta cell-specific *Dpy30* knockout
*Dpy30*-WT: *Dpy30* wild-type
H3K27ac: Histone H3 lysine 27 acetylation
H3K27me3: Histone H3 lysine 27 trimethylation
H3K4me1: Histone H3 lysine 4 monomethylation
H3K4me3: Histone H3 lysine 4 trimethylation
IHC: Immunohistochemistry
IP: Immunoprecipitation
OCR: Oxygen consumption rate
RBBP5: Retinoblastoma-binding protein 5
RNA-seq: RNA sequencing
SCENIC: Single-cell regulatory network inference and clustering
scRNA-seq: Single-cell RNA-seq
TSS: Transcription start site
UMAP: Uniform manifold approximation and projection
WDR5: WD repeat-containing protein 5
