# Supplementary material for: Methylation of histone H3 lysine 4 is required for maintenance of beta cell function in adult mice
Source: Diabetologia. 2023 Mar 13;66(6):1097–115. doi: 10.1007/s00125-023-05896-6 (PMC10163146; doi:10.1007/s00125-023-05896-6)
Supplement: Supplementary file 1 — (PDF 4873 kb) [file 125_2023_5896_MOESM1_ESM.pdf]

Electronic Supplementary Materials (ESM) for

Methylation of histone H3 lysine 4 is required for maintenance of beta cell function in adult mice

Ben Vanderkruk, Nina Maeshima, Daniel J. Pasula, Meilin An, Cassandra L. McDonald, Priya Suresh, Dan S. Luciani, Francis C. Lynn, Brad G. Hoffman

ESM Methods

Protein co-immunoprecipitation Immunoprecipitation of WDR5 was performed in triplicate, each with 750-900 dispersed islets pooled from 3 mice. First, nuclei were enriched from dispersed islet cells by incubation in nuclear isolation buffer (0.1% Triton X-100, 0.1% sodium deoxycholate, 1 mM PMSF, protease inhibitor cocktail (Roche)) for 10 min on ice and pelleting at 600 × *g* for 5 min at 4°C. Chromatin was solubilized in IP buffer (20 mM Tris pH 7.4, 150 mM NaCl, 1 mM EDTA, 5% (vol/vol) glycerol, 0.5% (vol/vol) Igepal ca-630, 5 mM $CaCl_2$, 1 mM PMSF, protease inhibitor cocktail) with MNase (New England Biolabs) for 10 min at room temperature. EDTA was added to 5 mM to stop MNase and samples were centrifuged at 14,000 × *g* for 5 min at 4°C. The protein concentration in the supernatant was measured by BCA assay (Life Technologies), and an equal mass of WT and KO nuclear lysate (~30 μg protein) was immunoprecipitated by 1 μg WDR5 (Bethyl, cat #A302-429A) or normal rabbit IgG (Millipore, cat #12-370) antibodies rotating overnight at 4°C. The next day, 10 μL protein A Dynabeads (Life Technologies) was added to the antibody:lysate mixture and rotated for 2 h at 4°C. Finally, bead:antibody:antigen complexes were washed 6 times in ice-cold IP buffer and resuspended in Laemmli sample buffer (2% (wt/vol) SDS, 10% (vol/vol) glycerol, 60 mM Tris pH 6.8, 1 mM NaF, 1 mM PMSF, EDTA-free protease inhibitor cocktail) with 5% (vol/vol) 2-mercaptoethanol for immunoblotting.

Immunoblotting Islets were lysed in Laemmli sample buffer for 5 min at 95°C and then vortexed at full speed for 10 to 20 s. Protein concentration was measured by BCA assay and then 2-mercaptoethanol was added to 5% (vol/vol), lysates were reboiled for 5 min, and then stored at –80°C. Ten micrograms of lysates were resolved in polyacrylamide gels, transferred to PVDF membranes, blocked for 1 h in 5% (wt/vol) skim milk powder in TBST (20 mM Tris pH 7.6, 150 mM NaCl, 0.1% (vol/vol) Tween-20) at room temperature and then probed overnight with primary antibodies diluted in 5% (wt/vol) BSA in TBST at 4°C. Membranes were washed for 10 min three times at room temperature with TBST, incubated with HRP-conjugated secondary antibodies for 1 h at room temperature in TBST, washed for 10 min three times at room temperature with TBST, and detected using ECL system with radiographic film. To normalise to H3, antibodies were stripped from membranes for 20 min at room temperature in mild stripping buffer (200 mM glycine, 0.1% (wt/vol) SDS, 1% (vol/vol) Tween-20, pH 2.2), washed twice with PBS and twice with TBST for 10 min at room temperature, then blocked and probed for H3.

Immunohistochemistry Whole pancreata from unperfused mice were fixed overnight at 4°C in 4% (wt/vol) formaldehyde in PBS then dehydrated through a graded ethanol, xylenes, and paraffin exchange system (overnight in 70% ethanol, 2× 30 min in 95% ethanol, 2× 30 min in 100% ethanol, 2× 30 min in xylenes, all at 4°C, and then 2× 1 h in melted paraffin wax at 60°C) before embedding in paraffin. Processed tissues were sectioned with a thickness of 5 μm. Sectioned tissues were rehydrated through graded xylene and ethanol exchange at room temperature (3× 5 min in xylenes, 2× 5 min in 100% ethanol, 5 min in 95% ethanol, 5 min in 70% ethanol, 10 min in water). Antigen retrieval was performed for 10 min at 95°C to 100°C in citrate buffer (10 mM citrate, 0.05% (vol/vol) Tween-20, pH 6.0), then slides were cooled to room temperature, washed with water, washed with PBS, blocked for 1 h using 5% (wt/vol) BSA in PBS at room temperature, probed overnight with primary antibodies diluted in 5% (wt/vol) BSA in PBS at 4°C, washed 3× 10 min at room temperature with PBS, probed for 1 h at room temperature with secondary antibodies and DAPI diluted in PBS, washed 3× 10 min at room temperature with PBS, and then mounted with ProLong Gold mounting solution (Life Technologies). Images were captured with a TCS SP8 confocal microscope (Leica, Germany) and quantified using CellProfiler v2 [1].

Chromatin immunoprecipitation-sequencing (ChIP-seq) The ULI-NChIP protocol [2] was used to generate ChIP-seq libraries in biological duplicate, with modifications. For *Pdx1-CreER*$^{Tg/0}$; *Dpy30*$^{+/+}$;

Rosa26$^{mTmG/+}$ and *Pdx1-CreER*$^{Tg/0}$; *Dpy30*$^{flox/flox}$; *Rosa26*$^{mTmG/+}$ mice, 100,000 EGFP+ tdTomato- islet cells were pooled with 50,000 *Drosophila* S2 cells by FACS. For *Lepr*$^{db/db}$ and *Lepr*$^{+/+}$ mice, 100,000 dispersed islet cells were counted by hemacytometer and pooled with 50,000 *Drosophila* S2 cells without sorting. Pooled cells were centrifuged at 600 × *g* for 5 minutes at 4°C, supernatant removed, and then cell pellets were flash-frozen and stored at -80°C for up to one month. Upon thawing, cells were permeabilized in nuclear isolation buffer containing 1 mM phenylmethylsulfonyl fluoride (PMSF) and EDTA-free protease inhibitor cocktail (Roche)) on ice. Chromatin was fragmented to mononucleosomes by micrococcal nuclease (MNase) (New England Biolabs) in digestion buffer (50 mM Tris pH 7.9, 5 mM $CaCl_2$, 1.5 mM DTT) for 7.5-minutes at 37°C, then quenched by addition of 0.1 volume of a solution containing 2% Triton X-100, 2% sodium deoxycholate, and 100 mM EDTA. Soluble chromatin was pre-cleared with Protein A Dynabeads (Thermo Fisher Scientific) pre-adsorbed with normal rabbit IgG (Millipore, 12-370) for 2-hours at 4°C rotating 9 rpm, and then rotated overnight at 4°C with 1 μg of H3K4me3 (Abcam, ab1012), 2 μg H3K4me1 (Abcam, ab8895), 1 μg H3K27ac (Active Motif, 39034), or 2 μg H3K27me3 (Millipore, 07-449) antibodies pre-adsorbed to 10 μL of Protein A Dynabeads (Thermo Fisher Scientific) in 150 μL of ChIP buffer (20 mM Tris pH 8.0, 2 mM EDTA, 150 mM NaCl, 0.2% Triton X-100, 1 mM PMSF, and EDTA-free protease inhibitor cocktail (Roche)). After washing the beads twice with ice-cold wash buffer (20 mM Tris pH 8, 150 mM NaCl, 2 mM EDTA, 1% (v/v) Triton X-100, 0.1% (w/v) SDS) and twice with ice-cold high-salt wash buffer (20 mM Tris pH 8, 500 mM NaCl, 2 mM EDTA, 1% (v/v) Triton X-100, 0.1% (w/v) SDS), DNA was eluted in elution buffer (100 mM $NaHCO_3$, 1% (w/v) SDS, 50 μg/mL RNase A) for 1-hour at 55°C, with addition of 1 μL 20 mg/mL proteinase K after the first 30-minutes and occasional manual inversion. DNA was purified using standard Phenol:chloroform:isoamyl alcohol extraction and ethanol precipitation and used for library preparation with the NEBNext Ultra II DNA Library Prep Kit for Illumina (New England Biolabs) with 9 (H3K4me1, H3K27me3) or 13 (H3K4me3, H3K27ac) amplification cycles. Indexed libraries were analyzed for size distribution using the Bioanalyzer High Sensitivity DNA chip (Agilent) and for concentration using the Qubit dsDNA HS Assay (Thermo Fisher Scientific). *Dpy30*-KO samples were sequenced on the NextSeq 500 platform (Illumina) for 2 × 78 nucleotide paired end reads and *Lepr*$^{db/db}$ samples on the NextSeq 2000 platform (Illumina) for 2 × 61 nucleotide paired end reads. Sequence data is available in the Gene Expression Omnibus (GEO) under accession number GSE181951.

ChIP-seq reads were aligned to concatenated mouse (GRCm38/mm10) and *Drosophila* (BDGP6.28) genome assemblies using Bowtie2 v2.3.4.1 with options '--very-sensitive --no-unal --no-discordant' [3]. Multi-mapped reads, reads with mapping quality MAPQ < 20, and suspected PCR-duplicates were discarded using Samtools [4]. To scale enrichment according to the *Drosophila* cell spike-ins, the fraction of remaining reads that mapped to the *Drosophila* genome was determined, and a scaling factor was calculated for each sample as *(minimum* Drosophila *read fraction per histone mark)/(sample* Drosophila *read fraction)*. After determining a scaling factor, each sample was downsampled to the lowest mapped read count per each histone mark using Samtools, and then reads mapped to the *Drosophila* genome were removed. For visualization, libraries were converted to bedgraph format using the calculated scaling factor in the '-scale' argument of BEDtools v2.26.0 genomecov [5]. Genome browser views were generated for merged replicates using Spark [6] and TSS profiles using Deeptools v3.4.2 [7]. Peak locations and breadth were determined using MACS2 v2.2.6 with parameters '--broad --nolambda' [8]. Peaks detected in only one biological replicate or that overlapped with high-background blacklisted regions defined by the ENCODE project [9] were excluded. Chromosomes X, Y, and M were also excluded from the analyses. DESeq2 [10] was used for differential peak width analysis using a significance cutoff of $P \leq 0.05$ (Wald test with Benjamini and Hochberg adjustment), and enrichment for differential breadth in peak quantiles was determined by one-sided Fisher's exact test. We defined TSS locations as the 5' end of the most abundant annotated transcript of each gene detected in our RNA-seq data. H3K4me3 peaks were assigned to genes for which they overlap the region ±1 kb of the TSS. For ESM Fig. 4b, H3K27ac peaks were assigned to the nearest TSS with no maximum distance. The null distribution of peak-to-gene associations was estimated by randomly permuting peak genomic locations corresponding 100 times using bedtools shuffle.

RNA-seq EGFP+ tdTomato- beta cells (70,000-226,000 per mouse, biological triplicate) were purified and counted by FACS and supplemented with a 10% spike-in of *Drosophila* S2 cells. *Drosophila* cells were included to detect global changes in cell RNA content [11]. The cells were sorted directly into Trizol LS Reagent (Thermo Fisher Scientific) and total RNA was extracted according to manufacturer's instructions.

Residual DNA was digested by Turbo DNase (Thermo Fisher Scientific) for 30-minutes at 37°C. Purified total RNA content was measured using the Qubit RNA HS assay (Thermo Fisher Scientific). Mature mRNA was enriched from 400 ng of total RNA per sample using the NEBNext Poly(A) mRNA Magnetic Isolation Module (New England Biolabs) and used to prepare libraries with the NEBNext Ultra II Directional RNA Library Prep Kit for Illumina (New England Biolabs) with 11 amplification cycles. Uniquely indexed libraries were quantified, pooled, and sequenced for 2 × 38 nucleotide reads as described above. Sequence data is available in the GEO under accession number GSE181951.

Sequenced reads were aligned to the concatenated mouse (GRCm38/mm10; Gencode vM24) and *Drosophila* (BDGP6.28; Ensembl release 99) genomes and transcriptomes using STAR v2.7.3a [12] with default settings. Transcript abundance was calculated using Salmon v1.4.0 [13] in alignment mode with seqBias and gcBias options. Gene counts and differential expression were calculated using DESeq2 [10]. Genes with fewer than two counts in any sample were filtered out and genes showing ≥ 2-fold difference in expression with $P \leq 0.01$ (Wald test with Benjamini and Hochberg adjustment for multiple comparisons) are considered differentially expressed. Genes showing <1.1-fold difference in expression are considered stable. Except in Fig. 3e, *Drosophila* transcripts were removed after abundance calculation; for Fig. 3e, *Drosophila* transcripts were used as control genes in the estimateSizeFactors function of DESeq2, and then removed.

Single cell RNA-seq Islets from one WT and one KO mouse were dissociated to single-cell suspensions as described above without FACS-enrichment. The cell suspensions were processed through the Chromium Single Cell 3' protocol using the Chromium Controller (firmware v4.0) with Reagent Kit v3.1 (10X Genomics) and Dual Index Kit TT Set A (10X Genomics) according to the manufacturer's instructions for a targeted 5,000-cell recovery. Total cDNA was amplified for 11-cycles, and then 13-cycles during index-ligation. cDNA concentrations were quantified by qPCR with the NEBNext Library Quant Kit for Illumina (New England Biolabs). Libraries were pooled and sequenced for 28-90 paired-end nucleotide reads to an average depth of ~70,000 mapped fragments per cell. Sequence data is available in the GEO under accession number GSE181951.

Cellranger (v5.0.0, 10X Genomics) was used to generate FASTQ files and demultiplex reads. Cell barcode detection, read mapping, quality filtering, and transcript counting were performed using Alevin (Salmon v1.4.0) [14] against the mouse protein-coding transcriptome (Gencode vM24), which we appended with the *Tdtomato* and *Egfp* sequences, as well as the mouse genome (GRCm38/mm10) as mapping decoys for selective alignment [15]. Additional cell quality filtering was performed using Seurat v4.1.2 [16] based on unique features (> 750 genes) and mitochondrial RNA content (< 20%). Putative doublets were excluded using DoubletFilter v2.0 [17]. Genes detected in < 3 cells were excluded from analysis. After quality filtering, the dataset comprised 3484 WT and 3685 KO cells. Filtered data were scaled and normalized using SCTransform v0.3.5 [18] with default parameters. PCA and UMAP dimensionality reduction using the first 60 principal components was used to cluster and visualize islet cell populations. Beta cell clusters (identified based on *Ins1/2* expression) from WT and KO samples were then merged, transformed, and clustered in UMAP space using 15 principal components. Slingshot v2.4.0 [19] was used for pseudotime analysis to rank cells along a continuous trajectory of transcriptome remodeling from beta cell cluster 1 to 2. Entropy scores are a modified Shannon entropy calculated using a published R function (ref [20]). Variance/mean ratios for each gene in each beta cell cluster was calculated from the raw RNA count, excluding genes detected in < 1 % of cells. Gene regulatory network inference was performed using SCENIC [21, 22]. For this, raw RNA counts from cells in beta cell clusters were exported in loom format using SCopeLoomR v0.13.0. A list of mouse transcription factors was downloaded from the cisTarget database (https://resources.aertslab.org/cistarget/). Co-expression modules were inferred using GRNBoost2 in a Docker Hub image of pySCENIC v0.12.0 [22]. This step was performed 100 times (with seed 1-100) and transcription factor/gene pairs identified in fewer than 80 iterations were discarded. The "importance" values of surviving pairs were averaged. The resulting high-confidence gene network was processed through pySCENIC's ctx and aucell functions using mouse transcription factor motif and target gene promoter annotations downloaded from the cisTarget database (motifs-v10nr_clust-nr.mgi-m0.001-o0.0.tbl, mm10_500bp_up_100bp_down_full_tx_clustered.genes_vs_motifs.rankings.feather, mm10_10kbp_up_10kbp_down_full_tx_clustered.genes_vs_motifs.rankings.feather) with default settings. The resulting loom file was loaded back into R. To plot the relative activity of each regulon in individual beta cells,

regulon AUCs were added to the Seurat object using CreateAssayObject() and scaled using ScaleData() commands. To compare the overall activity of each regulon between beta cell clusters, regulon specificity scores were generated using the calcRSS() command after down-sampling cells to equalize cell numbers in each cluster being compared.

ESM Methods References

1. Carpenter AE, Jones TR, Lamprecht MR, et al (2006) CellProfiler: Image analysis software for identifying and quantifying cell phenotypes. Genome Biol 7(10). https://doi.org/10.1186/gb-2006-7-10-r100
2. Brind'Amour J, Liu S, Hudson M, Chen C, Karimi MM, Lorincz MC (2015) An ultra-low-input native ChIP-seq protocol for genome-wide profiling of rare cell populations. Nat Commun 6:1–8. https://doi.org/10.1038/ncomms7033
3. Langmead B, Salzberg SL (2012) Fast gapped-read alignment with Bowtie 2. Nat Methods 9(4):357–359. https://doi.org/10.1038/nmeth.1923
4. Li H, Handsaker B, Wysoker A, et al (2009) The Sequence Alignment/Map format and SAMtools. Bioinformatics 25(16):2078–2079. https://doi.org/10.1093/bioinformatics/btp352
5. Quinlan AR, Hall IM (2010) BEDTools: A flexible suite of utilities for comparing genomic features. Bioinformatics 26(6):841–842. https://doi.org/10.1093/bioinformatics/btq033
6. Kurtenbach S, William Harbour J (2019) SparK: A Publication-quality NGS Visualization Tool. bioRxiv 1–4. https://doi.org/10.1101/845529
7. Ramírez F, Ryan DP, Grüning B, et al (2016) deepTools2: a next generation web server for deep-sequencing data analysis. Nucleic Acids Res 44(W1):W160–W165. https://doi.org/10.1093/nar/gkw257
8. Zhang Y, Liu T, Meyer CA, et al (2008) Model-based analysis of ChIP-Seq (MACS). Genome Biol 9(9). https://doi.org/10.1186/gb-2008-9-9-r137
9. Amemiya HM, Kundaje A, Boyle AP (2019) The ENCODE Blacklist: Identification of Problematic Regions of the Genome. Sci Rep 9(1):1–5. https://doi.org/10.1038/s41598-019-45839-z
10. Love MI, Huber W, Anders S (2014) Moderated estimation of fold change and dispersion for RNA-seq data with DESeq2. Genome Biol 15(12):1–21. https://doi.org/10.1186/s13059-014-0550-8
11. Taruttis F, Feist M, Schwarzfischer P, et al (2017) External calibration with Drosophila whole cell spike ins delivers absolute mRNA fold changes from human RNA seq and qPCR data. Biotechniques 62(2):53–61. https://doi.org/10.2144/000114514
12. Dobin A, Davis CA, Schlesinger F, et al (2013) STAR: Ultrafast universal RNA-seq aligner. Bioinformatics 29(1):15–21. https://doi.org/10.1093/bioinformatics/bts635
13. Patro R, Duggal G, Love MI, Irizarry RA, Kingsford C (2017) Salmon provides fast and bias-aware quantification of transcript expression. Nat Methods 14(4):417–419. https://doi.org/10.1038/nmeth.4197
14. Srivastava A, Malik L, Smith T, Sudbery I, Patro R (2019) Alevin efficiently estimates accurate gene abundances from dscRNA-seq data. Genome Biol 20(65). https://doi.org/10.1101/335000
15. Sarkar H, Zakeri M, Malik L, Patro R (2017) Towards selective-alignment: Bridging the accuracy gap between alignment-based and alignment-free transcript quantification. bioRxiv 1–7. https://doi.org/10.1101/138800
16. Stuart T, Butler A, Hoffman P, et al (2019) Comprehensive Integration of Single-Cell Data. Cell 177(7):1888-1902.e21. https://doi.org/10.1016/j.cell.2019.05.031
17. McGinnis CS, Murrow LM, Gartner ZJ (2019) DoubletFinder: Doublet Detection in Single-Cell RNA Sequencing Data Using Artificial Nearest Neighbors. Cell Syst 8(4):329-337.e4. https://doi.org/10.1016/j.cels.2019.03.003
18. Hafemeister C, Satija R (2019) Normalization and variance stabilization of single-cell RNA-seq data using regularized negative binomial regression. bioRxiv 1–15. https://doi.org/10.1101/576827
19. Street K, Risso D, Fletcher RB, et al (2018) Slingshot: cell lineage and pseudotime inference for single-cell transcriptomics. BMC Genomics 19(477):1–16
20. Kannan S, Farid M, Lin BL, Miyamoto M, Kwon C (2021) Transcriptomic entropy benchmarks stem cell-derived cardiomyocyte maturation against endogenous tissue at single cell level. PLoS Comput Biol 17(9):1–21. https://doi.org/10.1371/journal.pcbi.1009305
21. Aibar S, González-Blas CB, Moerman T, et al (2017) SCENIC: Single-cell regulatory network inference and clustering. Nat Methods 14(11):1083–1086. https://doi.org/10.1038/nmeth.4463
22. Van de Sande B, Flerin C, Davie K, et al (2020) A scalable SCENIC workflow for single-cell gene regulatory network analysis. Nat Protoc 15(7):2247–2276. https://doi.org/10.1038/s41596-020-0336-2

ESM Figures

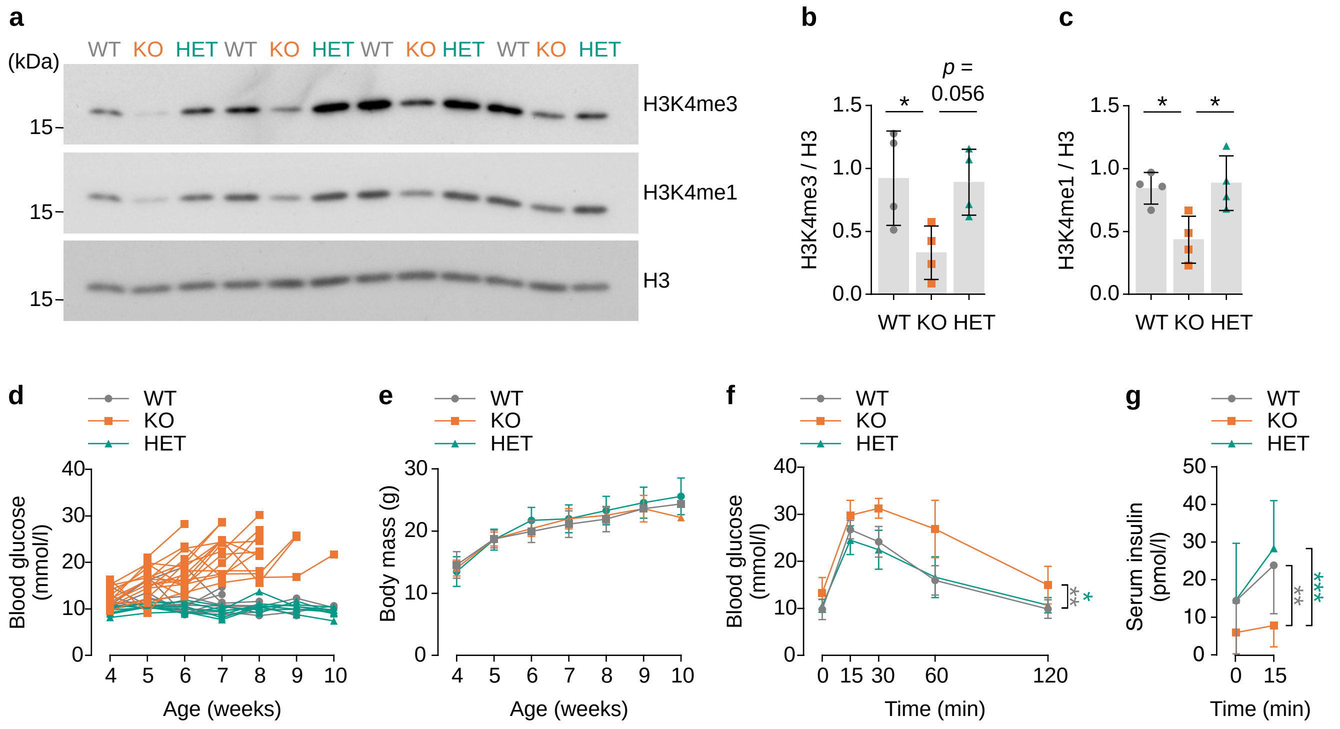

ESM Fig. 1 Deletion of *Dpy30* in mouse beta cells using *Ins1*^{Cre} results in reduction of H3K4 methylation, hyperglycemia, hypoinsulinemia and impaired glucose tolerance. (**a**) Immunoblots of H3K4me3, H3K4me1 and total histone H3 in islet lysate from 5-weeks-old *Dpy30*-WT, *Dpy30*-KO and *Dpy30*-HET mice. (**b**, **c**) Bar graphs showing band intensities of data in (**a**). *P* values were calculated using one-way ANOVA with Tukey's multiple comparisons correction (n=4). (**d**, **e**) Unfasted blood glucose concentration (**d**) and body mass (**e**) of *Dpy30*-WT, *Dpy30*-KO and *Dpy30*-HET mice between 4 to 10 weeks of age. Data are individual measurements with means (n=10 WT, n=15 KO, n=7 HET; however, tracking was stopped after 1 to 2 blood glucose reading ≥20 mmol/l). (**f**, **g**) Blood glucose (**f**) and serum insulin (**g**) levels during an IPGTT in 5-weeks-old *Dpy30*-WT, *Dpy30*-KO and *Dpy30*-HET mice. *P* values were calculated by comparison of KO versus HET (teal) or KO versus WT (grey) AUC's using one-way ANOVA with Tukey's multiple comparisons correction. *$p<0.05$, **$p<0.01$, ***$p<0.001$. HET, *Ins1*$^{Cre/+}$;*Dpy30*$^{flox/+}$. KO, *Ins1*$^{Cre/+}$;*Dpy30*$^{flox/flox}$. WT, *Ins1*$^{+/+}$;*Dpy30*$^{flox/flox}$

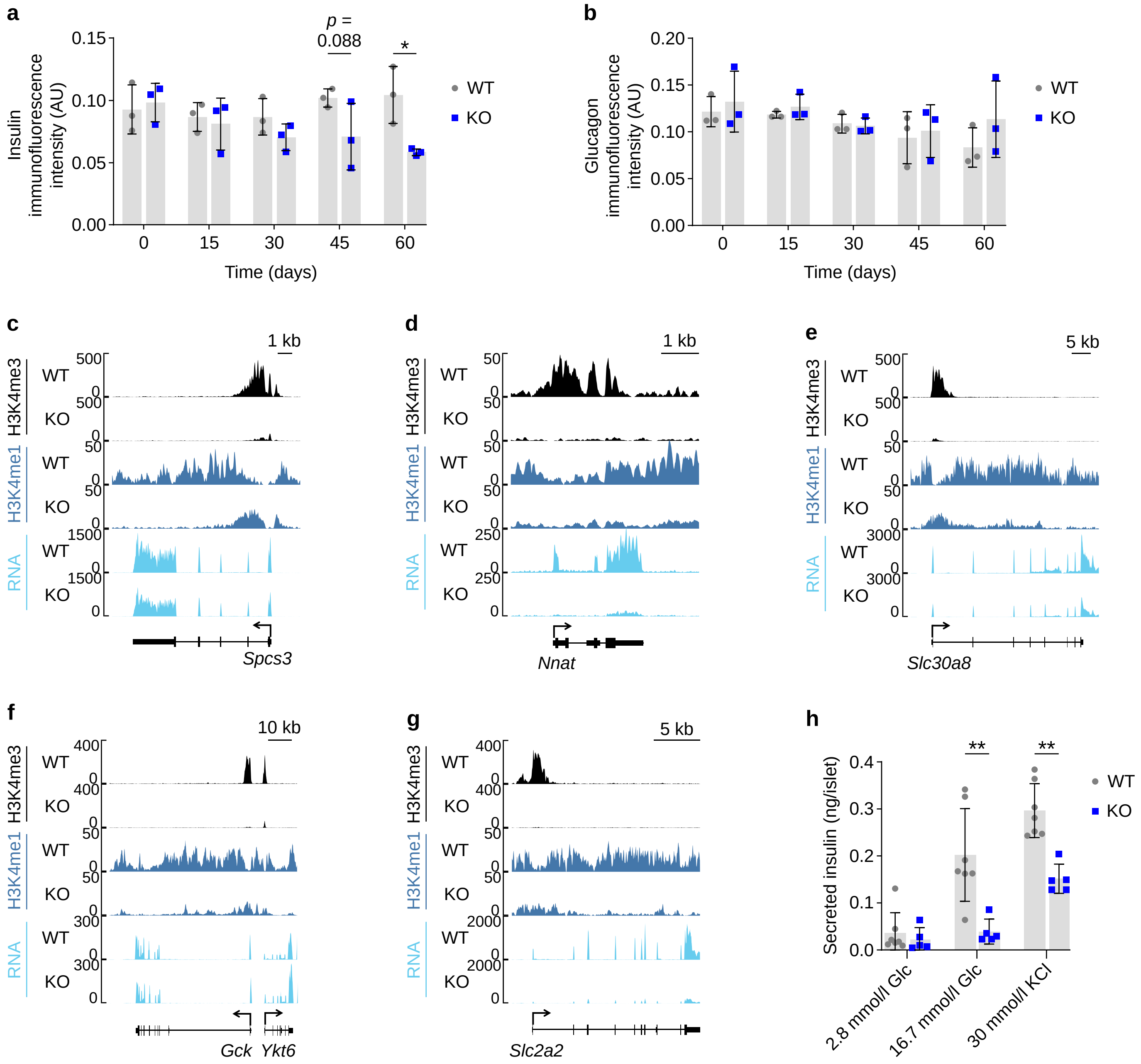

ESM Fig. 2 Genes involved in insulin production and glucose-induced activity are regulated by H3K4me3. (**a**, **b**) Mean immunofluorescence intensity of insulin (**a**) and glucagon (**b**) in *Dpy30*-WT and *Dpy30*-KO islets at the indicated days after tamoxifen administration (n=3). (**c-g**) Genome browser views of H3K4me3, H3K4me1, and RNA in *Dpy30*-WT and *Dpy30*-KO chromatin at selected genes. (**h**) Bar graph showing insulin secretion from *Dpy30*-WT and *Dpy30*-KO islets during static in vitro stimulation with glucose and KCl solutions (n=7 WT, n=5 KO). *P* values were calculated using two-way ANOVAs with Benjamini-Hochberg correction. *$p<0.05$, **$p<0.01$, AU, arbitrary units; Glc, glucose

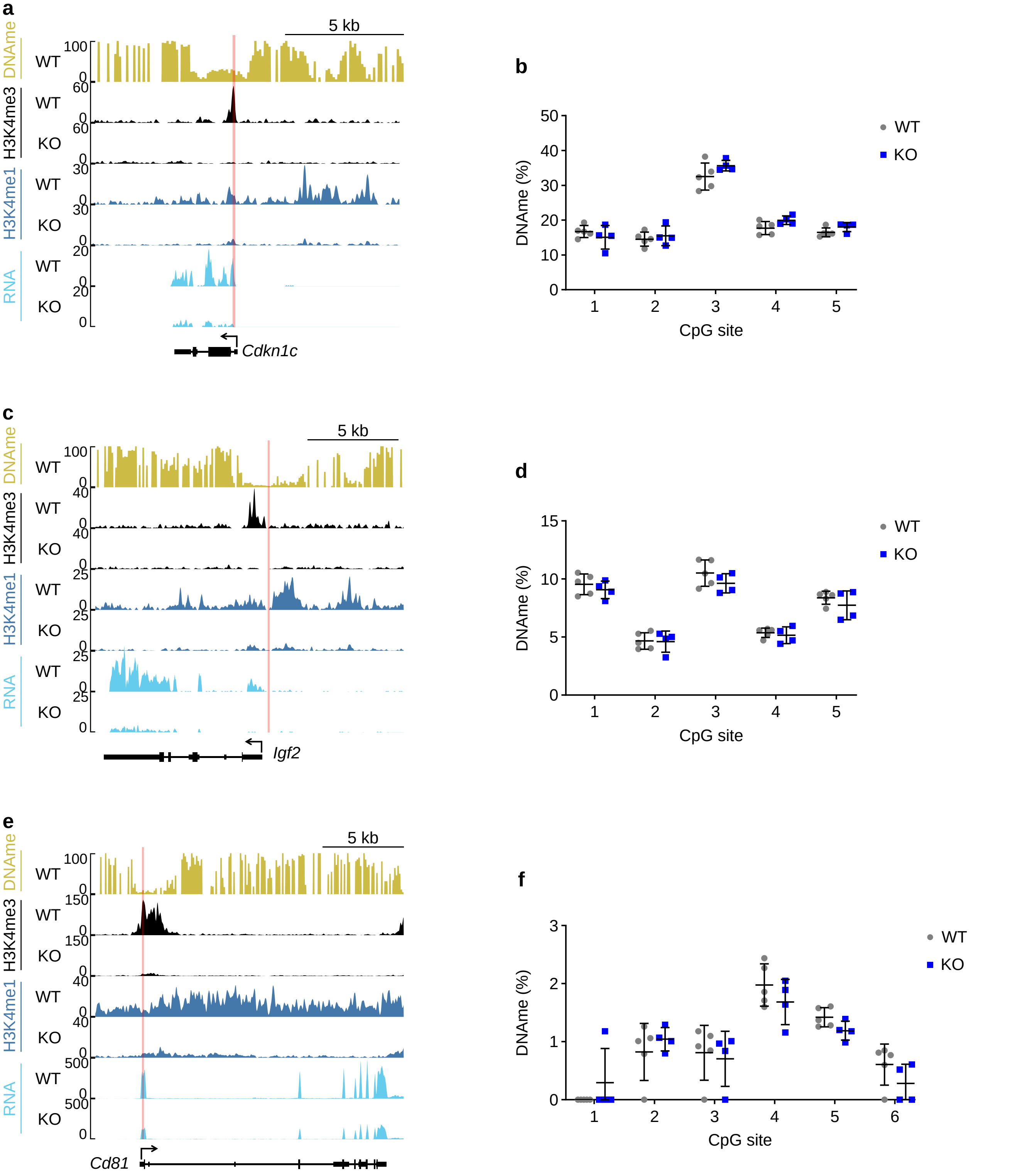

ESM Fig. 3 Loss of H3K4 methylation did not lead to gain of DNA methylation at three selected loci. (**a**) Genome browser view of DNAme, H3K4me3, H3K4me1, and RNA at the *Cdkn1c* gene locus. The DNAme track shows average CpG cytosine methylation (%) in 100 bp windows and was downloaded from GSE68618 [55]. The locus targeted for pyrosequencing is highlighted in red. (**b**) Average DNAme of CpG sites in the red locus of (**a**) in *Dpy30*-WT and *Dpy30*-KO islets. (**c**, **d**) The same analysis as in (**a**) and (**b**), respectively, at the *Igf2* gene locus. (**e**, **f**) The same analysis as in (**a**) and (**b**), respectively, at the *Cd81* gene locus (n=5 WT, n=4 KO). Data are p>0.05 for change in methylation at any site using two-way ANOVA with Benjamini -Hochberg correction

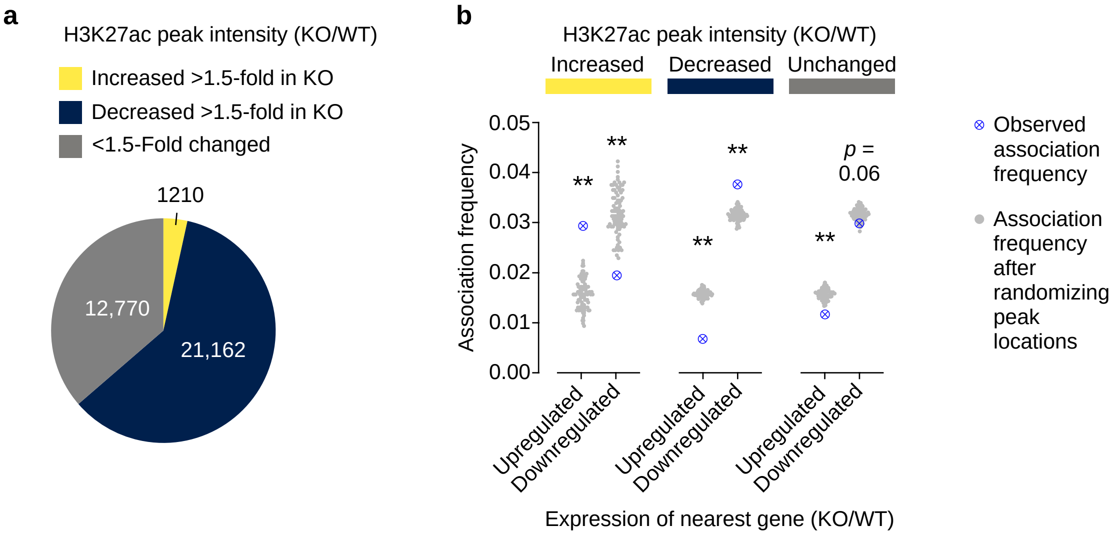

ESM Fig. 4 H3K27ac peak dynamics are linked to gene expression dynamics in *Dpy30*-KO cells. (**a**) Pie chart showing the number of H3K27ac peaks that are increased >1.5-fold, decreased >1.5-fold or <1.5-fold changed for enrichment of H3K27ac in *Dpy30*-KO vs *Dpy30*-WT chromatin. (**b**) Association frequency of H3K27ac peaks classified in (**a**) with genes that are upregulated or downregulated in *Dpy30*-KO cells. Blue circled x shapes show the observed association frequency. Grey dots show the association frequency after randomizing the genomic location of peaks 100 times. For example, the left-most column shows that roughly 3% of H3K27ac peaks that increase in intensity are closest to genes that are upregulated; if H3K27ac peaks are randomly distributed in the genome, roughly 1 to 2% of H3K27ac peaks that increase in intensity are closest to genes that are upregulated. *P* values were calculated using permutation tests. ***p*<0.01

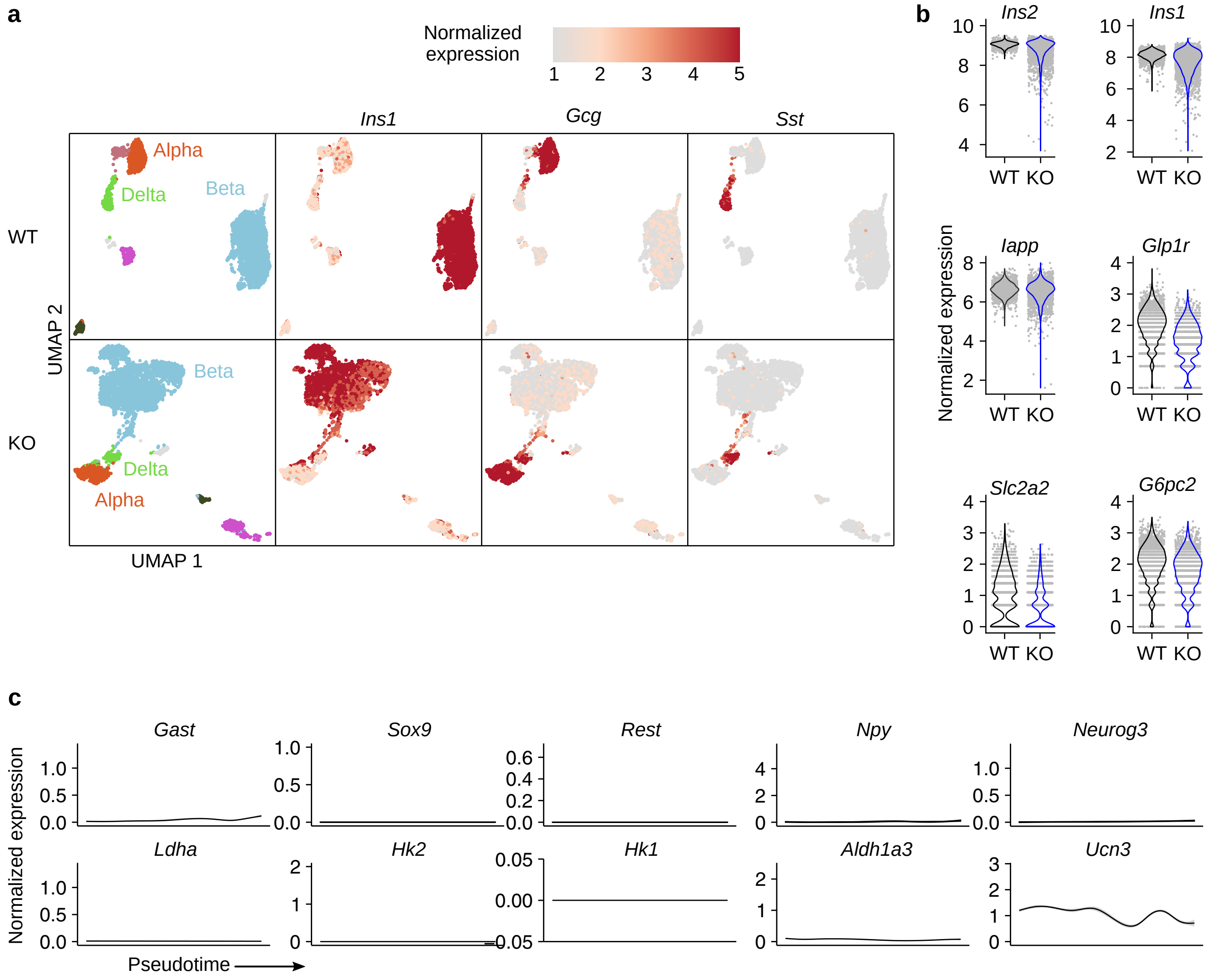

ESM Fig. 5 *Dpy30*-KO beta cells do not activate genes associated with dedifferentiated beta cells. (**a**) UMAP visualization of islet cell transcriptomes from a *Dpy30*-WT and a *Dpy30*-KO mouse. Markers of beta cells (*Ins1*), alpha cells (*Gcg*) and delta cells (*Sst*) are shown as a colour gradient (n=3484 WT cells, n=3684 KO cells). (**b)** Violin plots showing expression *Ins1*, *Ins2*, *Iapp*, *Glp1r*, *Slc2a2* and *G6pc2* in *Dpy30*-WT and *Dpy30*-KO beta cells (n=2170 WT cells, n=2707 KO cells). (**c**) Lowess curves of gene expression of the indicated maturity and immaturity genes in beta cells along pseudotime defined in Fig. 6c.

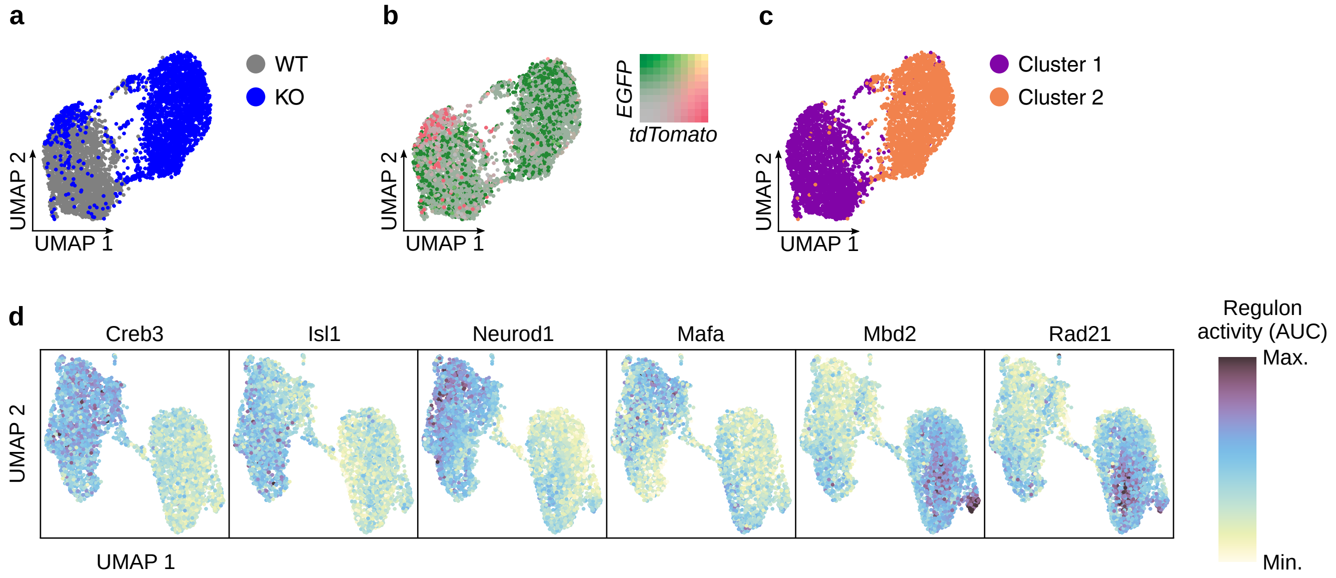

ESM Fig. 6 SCENIC analysis. (**a**–**c**) Beta cells embedded in UMAP space based on their regulon activities and coloured according to the genotype of the donor mouse (**a**), expression of *tdTomato*, *EGFP*, and their overlap (**b**) and the beta cell cluster it belongs to in UMAP space based on cell transcriptomes in Fig. 6a (**c**). (**d**) Beta cells embedded in transcriptome UMAP space as in Fig. 6a and coloured according to the regulon activity of selected transcription factors. AUC, area under the curve

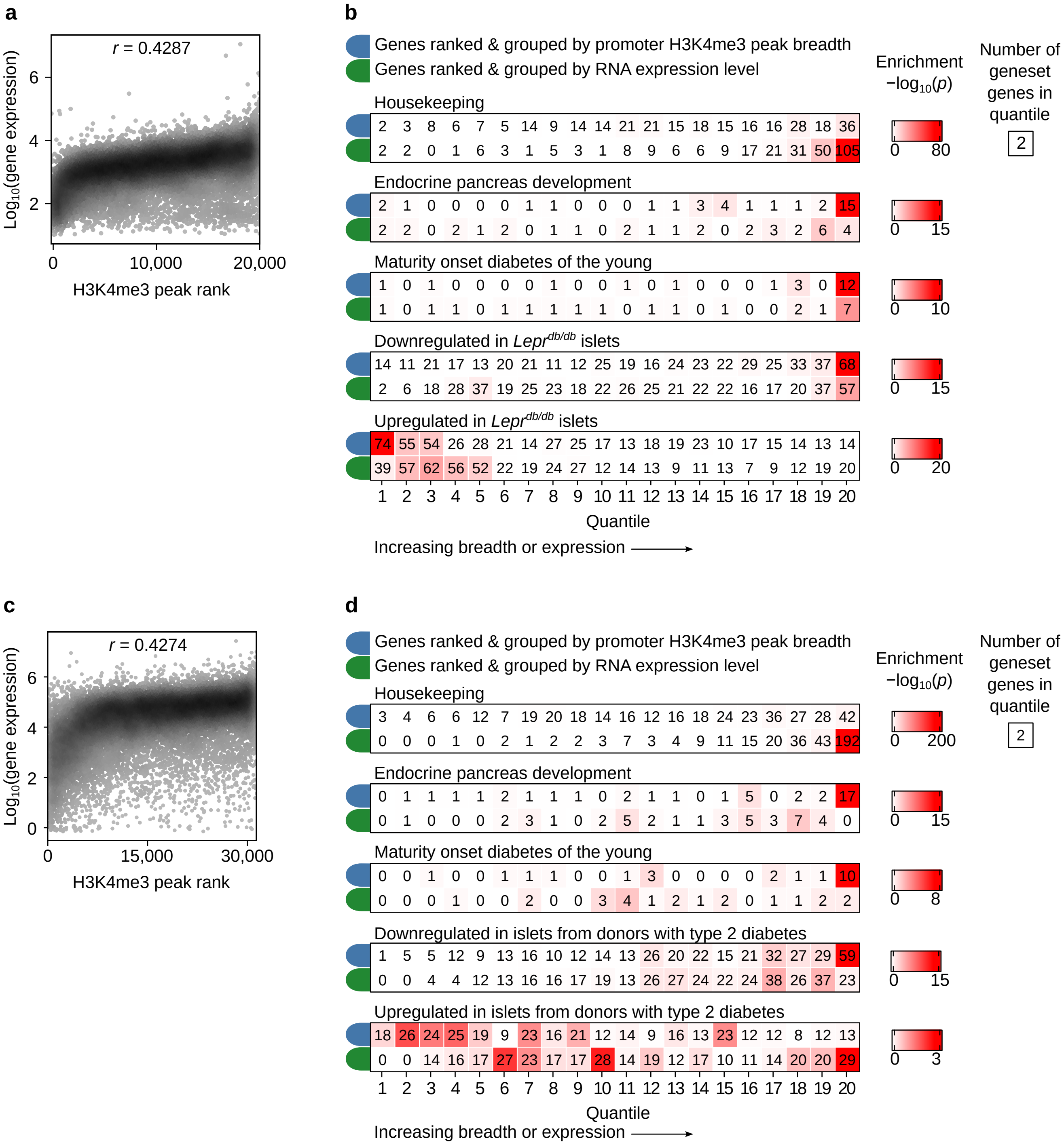

ESM Fig. 7 H3K4me3 peak breadth stratifies beta cell gene sets more effectively than gene expression level. (**a**) H3K4me3 peaks ranked by increasing breadth (x axis; same as Fig. 7a) plotted against RNA expression of the associated gene (y axis) in beta cells from *Dpy30*-WT mice. r indicates Spearman's rank correlation coefficient. (**b**) Gene set overrepresentation analysis. All genes expressed in mouse beta cells are ranked and grouped by H3K4me3 peak breadth from narrow to broad (blue) or ranked and grouped by gene expression level from low to high (green). The number in each square indicates the number of genes of each geneset matching to each quantile. Enrichment *p* values were calculated using one-sided Fisher's exact tests. *Lepr*$^{db/db}$ RNA-seq data is from Neelankal et al [53]. (**c**, **d**) Same analysis as in (**a**) and (**b**), respectively, in human, using ChIP-seq data from Bramswig et al [52] and RNA-seq data from Fadista et al [51]

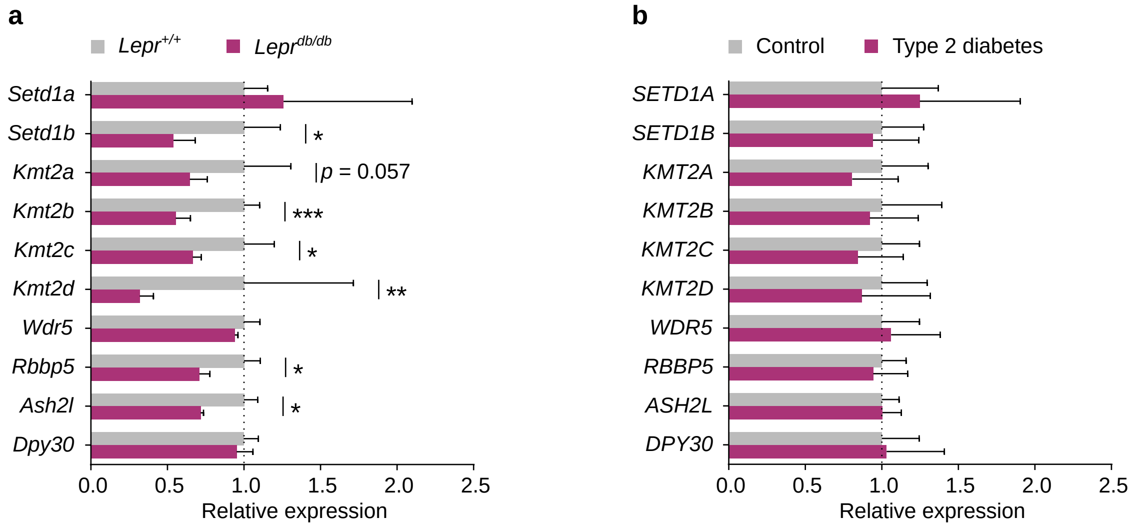

ESM Fig. 8 RNA expression of COMPASS factors in islets from mice and humans with diabetes. (**a**, **b**) Relative expression of COMPASS catalytic subunit genes and core noncatalytic subunit genes in islets from *Lepr*$^{+/+}$ and *Lepr*$^{db/db}$ mice (n=3) (**a**) or in islets from human donors with or without type 2 diabetes (n=51 control, n=11 type 2 diabetes) (**b**). *Lepr*$^{db/db}$ RNA-seq data is from Neelankal et al [53] and human RNA-seq data is from Fadista et al [51]. *P* values were calculated using Wald tests with Benjamini-Hochberg correction. *$p<0.05$, **$p<0.01$, ***$p<0.001$

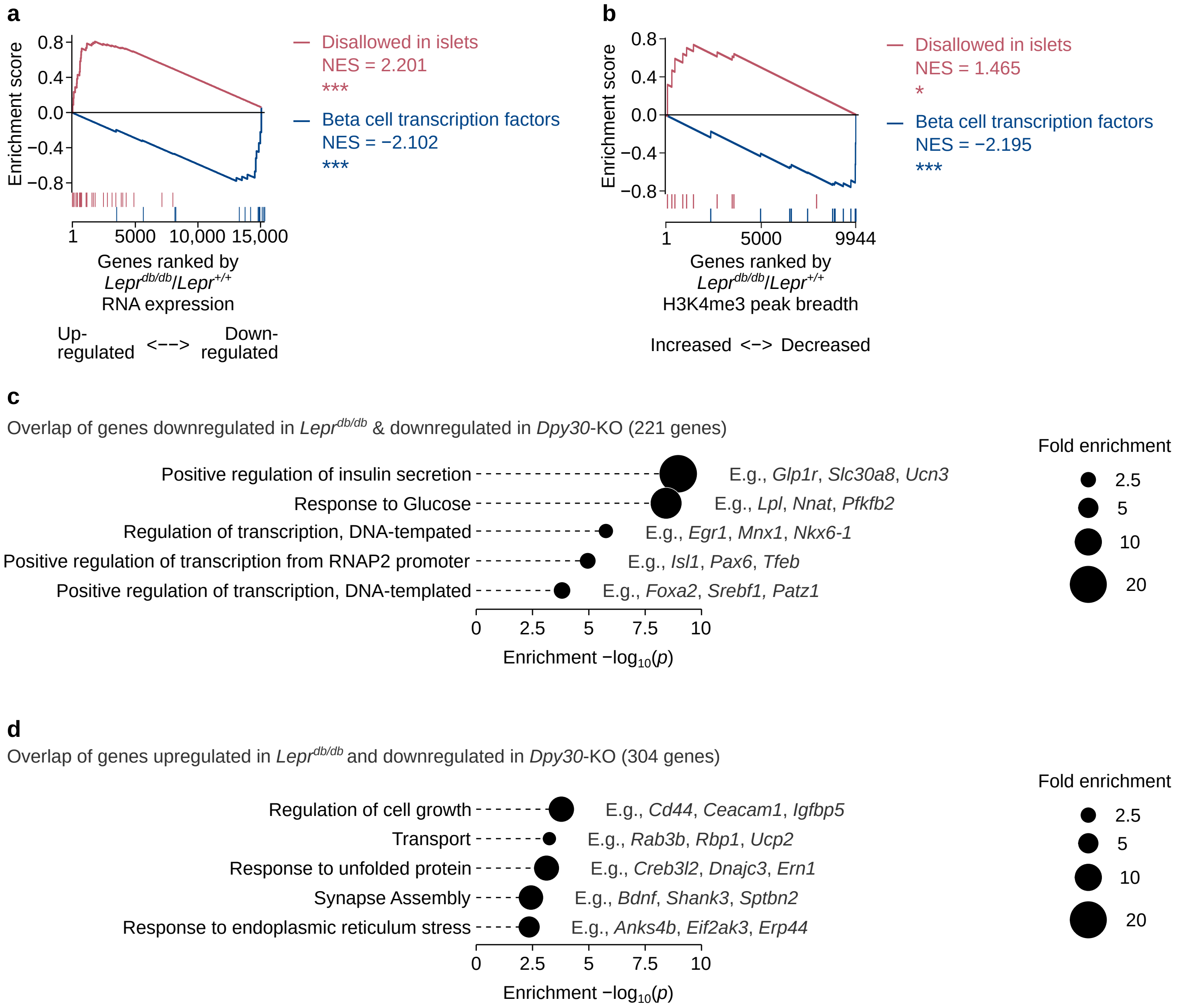

ESM Fig. 9 Enrichment analyses for gene expression in *Lepr*$^{db/db}$ islets. (**a**, **b**) Running enrichment plots of genes disallowed in islets (red) and mature beta cell transcription factor genes (blue) in islets from *Lepr*$^{db/db}$ vs *Lepr*$^{+/+}$ mice. In (**a**), genes are ranked by differential gene expression. In (**b**), genes are ranked by differential H3K4me3 peak breadth. *P* values were calculated using permutation tests. (**c**, **d**) Overrepresentation analysis of Gene Ontology: biological process terms in genes that were dysregulated in *Lepr*$^{db/db}$ and *Dpy30*-KO mice models. Panel (**c**) shows biological processes enriched in genes that were downregulated in both models, as determined by the RRHO2 package [73], and corresponding to the upper right quadrant of Fig. 7l. Panel (**d**) shows biological processes enriched in genes that were upregulated in *Lepr*$^{db/db}$ and downregulated in *Dpy30*-KO, as determined by the RRHO2 package [73], and corresponding to the lower right quadrant of Fig. 7l. Gene set enrichment and *p* values were calculated using DAVID v.6.8 [82]. The top five terms ranked by *p* values are shown. *p<0.05, ***p<0.001. NES, normalized enrichment score
